# Integrating agro-physiological traits and yield performance in soybean (*Glycine max* L.) resistance to fall armyworm (*Spodoptera frugiperda*) through genotype analysis

**DOI:** 10.7717/peerj.20753

**Published:** 2026-02-04

**Authors:** Anna Satyana Karyawati, Demas Dharmawan, Himma Rahmadillah, Budi Waluyo, Vina Mafazatul Ula

**Affiliations:** Agronomy, Brawijaya University, Malang, Indonesia

**Keywords:** Soybean resistance lines, Pest-resilient crops, Sustainable pest management, *Spodoptera litura* adaptation

## Abstract

Fall armyworm (*Spodoptera frugiperda*) is a major invasive pest threatening soybean production, and identifying resistant genotypes is essential for sustainable crop protection. This study evaluated 36 soybean genotypes for their resistance to *S. frugiperda* based on leaf damage intensity and frequency, resistance classification, morphophysiological traits, and yield components. The research was conducted under field conditions using a randomized complete block design (RCBD) with three replications. Armyworm damage was assessed at 36 and 41 days after planting (DAP), and all quantitative data were analyzed using ANOVA followed by the Scott–Knott test at a 5% significance level. Data were presented as mean ± standard deviation (SD). Significant variation was evident among genotypes, with attack intensity at 41 DAP ranging from 18.31 ± 10.64% to 61.20 ± 11.39%, and attack frequency from 33.38 ± 16.92% to 95.49 ± 5.35%. Based on mean intensity values, one genotype was categorized as strongly resistant (SR) (UB 2), four as resistant (R) (UBASK24, UBASK35, GBG, TGM), and a broader set as moderately resistant (MR), while highly susceptible (HS) genotypes included UBASK15, UBASK62, UBASK64, UBASK32, UBASK36, and UBASK14. Morphophysiological traits exhibited wide variation, including leaf trichome density (13.56–42.11 trichomes 0.25 cm^−2^), plant height (38.42–78.67 cm), and flowering time (31–47 DAP), while yield traits identified TGM, UBASK35, UBASK24, and GBG as the highest-performing genotypes. Overall, UB 2, UBASK24, UBASK35, GBG, and TGM are promising parental candidates for breeding soybean resistance against *S. frugiperda*, integrating strong resistance expression with favorable agronomic performance.

## Introduction

Soybean (*Glycine max* (L.) Merr.) plays a pivotal role as a global source of protein and vegetable oil, making it a cornerstone of food security and industrial applications. As a primary dietary staple in many developing countries, particularly in regions where plant-based protein dominates, soybean plays a crucial role in ensuring nutritional sufficiency for millions of people. Its economic importance is also evident, as it supports the livelihoods of farmers and contributes significantly to agricultural industries worldwide (Sasane et al., 2018). However, soybean productivity cultivation faces escalating challenges from biotic stressors, particularly infestations of *Spodoptera frugiperda* (fall armyworm), a highly destructive pest capable of causing yield losses of up to 80% in India (Prasad et al., 2021).

Conventional pest control strategies predominantly rely on synthetic pesticides, which although effective in the short term, pose significant ecological and economic drawbacks (Yifei et al., 2018). Overuse of chemical pesticides has led to pest resistance, resurgence, and non-target environmental contamination, threatening biodiversity and human health. According to global pesticide usage data, agriculture contributes to over two million tons of pesticide applications annually, exacerbating greenhouse gas emissions and soil degradation (Zhang et al., 2018). The environmental and economic costs of such dependence underscore the urgency for sustainable pest management strategies.

The most durable, economically and environmentally friendly management of this pest is developing soybean varieties with genetic resistance. By leveraging genetic and agro-physiological traits, these varieties can reduce reliance on chemical inputs while enhancing ecological resilience. Advances in plant breeding and integrated pest management offer innovative pathways to achieve productivity and sustainability simultaneously. However, achieving this balance remains challenging, as incorporation of resistance traits often involve trade-offs with yield performance. Understanding these dynamics is critical for developing soybean varieties that are both resilient to pests attack and highly productive (Thomas & Tabanca, 2025).

Recent advances have emphasized the importance of integrating genotype-specific responses with agronomic performance to develop soybean cultivars that are both pest-resistant and high-yielding. Morphological and biochemical traits such as leaf trichome density, enzyme activity, and stress-related gene expression have been associated with resistance to *S. frugiperda* (Shafi & Kariyat, 2025). Incorporating these traits into breeding strategies offers a promising pathway toward resilient and productive cultivars, especially under variable agroecological conditions. For example, Grobogan variety is recognized for its pods that are resistant to shattering at maturity, which helps minimize post-harvest losses and improve harvesting efficiency. Argopuro exhibits strong tolerance to several major pests, including pod-sucking insects, bean fly, armyworms, and Soybean Mosaic Virus (SMV). Anjasmoro demonstrates moderate resistance to soybean rust, a critical fungal disease that significantly reduces soybean productivityalso has adaptability to dryland and acidic soils. Tanggamus provides additional benefits, including resistance to lodging, which ensures better plant structure and easier harvesting. It also exhibits moderate resistance to soybean rust and is well adapted to dryland and acidic soils. UB1 and UB2 are notable for their tolerance to water stress, offering resilience in regions with irregular water availability or drought conditions.

This study investigates the resistance of 36 soybean genotypes, comprised of 30 hybrid lines and six parental varieties, against *S. frugiperda* infestations. Key agro-physiological traits, including leaf trichome density and yield components, were assessed to identify superior genotypes that integrate pest resistance with optimal yield traits.

Genotype-by-trait biplot analysis and path analysis were employed to unravel the complex relationships between resistance mechanisms and reproductive success (Patu et al., 2023). The findings aim to contribute to sustainable soybean production systems by reducing pesticide dependency and promoting eco-friendly pest management strategies. It is hypothesized that resistance to *Spodoptera frugiperda* in soybean results from the combined effect of multiple agro-physiological traits, including but not limited to trichome density and yield-related components.

By addressing the dual goals of productivity and sustainability, this research aligns with global efforts to achieve the United Nations’ Sustainable Development Goals (SDGs), particularly SDG 2 (Zero Hunger) and SDG 12 (Responsible Consumption and Production) (Singh, Sreenivasulu & Prasad, 2022). The implications extend beyond soybean cultivation, offering valuable insights into sustainable agricultural practices that advance ecological and economic resilience in the face of climate change.

## Materials and Methods

### Study site

The study was conducted from February to May 2022 at the Research Center for Legumes and Tuber Crops (BSIP AKABI) in Kendalpayak, Pakisaji, Malang Regency, Indonesia (8°2′51″ S, 112°37′33″ E). The site was located at an altitude of 445 m above the sea level, with maximum and minimum temperatures of 30 °C and 17.5 °C, respectively, and an average annual rainfall of 2,191 mm (Badan Pusat Statistik Kota Malang [BPS], 2022).

### Plant materials and treatments

The experiment utilized 30 soybean genotypes derived from crosses (UBASK16 (UB 2 × AJM), UBASK13 (AGP × AJM), UBASK14 (GBG × AJM), UBASK12 (TGM × AJM), UBASK15 (UB 1 × AJM), UBASK61 (AJM × UB 2), UBASK63 (AGP × UB 2), UBASK64 (GBG × UB 2), UBASK62 (TGM × UB 2), UBASK65 (UB 1 × UB 2), UBASK31 (AJM × AGP), UBASK36 (UB 2 × AGP), UBASK34 (GBG × AGP), UBASK32 (TGM × AGP), UBASK35 (UB 1 × AGP), UBASK41 (AJM × GBG), UBASK46 (UB 2 × GBG), UBASK43 (AGP × GBG), UBASK42 (TGM × GBG), UBASK45 (UB 1 × GBG), UBASK21 (AJM × TGM), UBASK26 (UB 2 × TGM), UBASK23 (AGP × TGM), UBASK24 (GBG × TGM), UBASK25 (UB 1 × TGM), UBASK51 (AJM × UB 1), UBASK56 (UB 2 × UB 1), UBASK53 (AGP × UB 1), UBASK54 (GBG × UB 1), UBASK52 (TGM × UB 1)) and six parental genotypes (Anjasmoro (AJM), Argopuro (AGP), Grobogan (GBG), Tanggamus (TGM), UB 1, UB 2). Other materials included NPK 16:16:16 fertilizer, pesticides, cow manure, soil, water, and larvae of *Spodoptera frugiperda*.

### Experimental design

A randomized complete block design (RCBD) was employed in this study. It consisted of 36 entries, comprising 30 cross-derived genotypes and six parental genotypes, with each replicated three times, thus resulting in 108 experimental units. Each plot contained 12 pots, with a total of 1,296 pots and 2,592 plants. Each pot was filled with a soil-manure mixture (3:1) and planted with three seeds, which was later thinned to two plants per pot. The pots were arranged directly in the field to allow exposure to natural environmental conditions while maintaining individual plant units. The use of RCBD with pot arrangements in field conditions has also been employed in previous studies assessing insect resistance in soybean genotypes, allowing for effective control of environmental variability while maintaining pest exposure (Sarjan, Fathiyani & Yassi, 2021). The present study follows a similar framework with modifications tailored to evaluate both vegetative and yield responses under controlled larval pressure.

### Pest inoculation and monitoring

Third instar larvae of *Spodoptera frugiperda* were inoculated 32 days after sowing (DAS), coinciding with the flowering stage of the soybean plants. Four larvae were introduced into each pot, each containing a single soybean plant, resulting in a larval density of four larvae per plant. To prevent larval escape and protect them from natural predators, each plant was enclosed within an individual mesh cage (2 m × 2 m) constructed with fine netting. No chemical insecticides or biological control agents were applied during the experimental period to ensure that observed feeding damage was solely attributable to the introduced larvae. Larval feeding damage was monitored daily to evaluate plant resistance levels.

### Data collection and parameter observation

For all destructive and non-destructive observations, including yield parameters, five plants per replication (15 plants per genotype) were randomly selected and tagged at early vegetative stages to ensure uniform monitoring until harvest. These representative samples formed the basis for a comprehensive data collection protocol, which was categorized as follows:

1. Destructive observations
  a. Leaf trichome density: Trichome density was assessed destructively at 28 DAS. Fully expanded leaves were collected, specifically the third or fourth leaf from the apex during the reproductive R1 phase (beginning of flowering). Trichomes on the abaxial surface were observed under a microscope, and their density per 0.25 cm^2^ was calculated (Hamawaki et al., 2012).
  b. Leaf area: Measurements were taken twice: 7 days before pest inoculation and 7 days after inoculation. Leaf samples were analyzed using a leaf area meter (LAM) to determine the reduction in leaf area due to pest damage (Nandan et al., 2022).
2. Non-destructive observations
  a. Plant height: Plant height was measured weekly from 14 DAS until harvest. The measurement was taken from the base of the stem to the growing tip using a measuring tape.
  b. Leaf count: The number of fully formed trifoliate leaves was recorded weekly from 14 DAS to harvest. This provided insights into growth dynamics and the effects of pest attack.
  c. Attack intensity: The severity of damage caused by *Spodoptera frugiperda* was assessed at 36 DAS and 41 DAS using a damage scale of I–X: I = 0–10%, II = >10–20%, III = >20–30%, IV = >30–40%, V = >40–50%, VI = >50–60%, VII = >60–70%, VIII = >70–80%, IX = >80–90%, and X = >90–100%. This scoring method was adapted from Sarjan, Fathiyani & Yassi (2021) to reflect the characteristic defoliation caused by chewing insects. and calculated as follows:
(i) $${\rm I}\left( { \rm \% } \right){\rm = }{{\sum {\rm (ni\; \times vi)}} \over {{\rm N\; \times Z}}}{\; \times 100\; }$$
    where *ni* is number of leaves in each damage category, *vi* is score for each category, *N* is total leaves observed, and *Z* is maximum damage score.
  d. Pest attack frequency: It was calculated as the percentage of affected leaves relative to the total number of leaves observed at 36 DAS and 41 DAS:
(ii) $${\rm F}\left( {\rm \% } \right){ = \; }{{\rm X} \over {\rm Y}}{ \; \times 100 }$$
    where *X* is number of damaged leaves, and *Y* is total leaves observed. Although originally applied to assess seed damage by a sucking pest, the method by Chen et al. (2024) was adapted to evaluate leaf-level feeding injury caused by a chewing insect. In this study, the formula was applied by quantifying the percentage of damaged leaves, which reflects foliar feeding intensity characteristic of *S. frugiperda* infestation.
3. Yield parameters
  a. Days to maturity: The time from sowing to 95% pod maturity, which was marked by a change in pod color to brown, was recorded.
  b. Number of productive branches: Productive branches were counted post-harvest based on the bearing pods.
  c. Fertile nodes: The number of nodes that produced pods was recorded after harvest.
  d. Total and filled pods: The total number of pods per plant, including filled and unfilled pods, was counted post-harvest. Filled pods were further differentiated by counting pods containing seeds.

### Statistical analysis

All data were subjected to analysis of variance (ANOVA) at a 5% significance level using DSAASTAT software. Prior to analysis, assumptions of normality and homoscedasticity were tested using the Shapiro-Wilk and Levene’s tests, respectively. All quantitative data were expressed as mean ± standard deviation (SD), and genotype means were separated using the Scott–Knott clustering test at a 5% significance level. This method controls for the family-wise error rate by clustering means into statistically distinct groups, thereby minimizing type I error without the need for additional correction procedures. In addition, path analysis was conducted to determine the direct and indirect effects of various growth parameters on yield components, providing a comprehensive understanding of the relationships among the observed traits. Standardized regression coefficients were used to partition the total correlations into direct and indirect effects. The analysis was carried out using RStudio version 4.3.2, ensuring robust and reproducible statistical computation.

To evaluate genetic and phenotypic similarities among genotypes, a dendrogram was constructed based on hierarchical clustering analysis. The standardized data were subjected to Euclidean distance calculation, and clustering was performed using the unweighted pair-group method with arithmetic mean (UPGMA). The dendrogram, created using R Studio version 4.3.2, visually represented the genetic relationships and grouping patterns among the soybean genotypes, providing valuable insights for selection and breeding strategies.

## Results

### Average intensity of armyworm attacks on soybean genotypes

Observations at 36 and 41 days after planting (DAP) showed variation in armyworm (*Spodoptera frugiperda*) attack intensity among soybean genotypes (Table 1). The intensity of *Spodoptera frugiperda* attacks at 36 days after planting (DAP) showed no statistically significant separation among genotypes, as all entries were grouped into the same Scott–Knott category (group a, indicating the higher-intensity cluster). Despite the uniform grouping, numerical variation was evident. Several genotypes experienced relatively high attack intensity, including UBASK15 (54.70 ± 22.22), UBASK32 (51.76 ± 19.19), UBASK36 (49.07 ± 29.08), UBASK64 (47.61 ± 8.34), and UBASK23 (49.64 ± 10.70). Meanwhile, genotypes such as UB 2 (13.16 ± 10.83), UBASK35 (20.57 ± 13.31), GBG (22.79 ± 6.37), UBASK43 (25.16 ± 12.07), and UBASK24 (30.09 ± 7.57) showed lower numerical damage intensities (Table 1), though these differences were not statistically significant at this early developmental stage. The variability in resistance is likely due to genetic factors, including antixenosis, antibiosis, and tolerance, which influence pest feeding behavior, survival, and plant recovery (Souza et al., 2021).

**Table 1:** Average intensity of armyworm (*Spodoptera frugiperda*) attacks on 36 soybean genotypes at 36 and 41 days after planting (DAP).

No.	Genotype	Non-selected (%)	
		36 DAP	41 DAP
1.	UBASK12	34.30 ± 7.83 a (IV)	41.99 ± 9.17 a (V)
2.	UBASK13	28.88 ± 13.06 a (III)	32.52 ± 12.55 b (IV)
3.	UBASK14	39.74 ± 6.89 a (IV)	56.58 ± 9.64 a (VI)
4.	UBASK15	54.70 ± 22.22 a (VI)	57.70 ± 11.03 a (VI)
5.	UBASK16	47.92 ± 14.80 a (V)	37.04 ± 8.08 b (IV)
6.	UBASK21	30.82 ± 6.57 a (III)	30.78 ± 15.66 b (III)
7.	UBASK23	49.64 ± 10.70 a (V)	30.93 ± 6.27 b (III)
8.	UBASK24	30.09 ± 7.57 a (III)	19.43 ± 1.29 b (II)
9.	UBASK25	42.94 ± 9.56 a (V)	25.96 ± 5.25 b (III)
10.	UBASK26	37.61 ± 12.98 a (IV)	27.05 ± 14.06 b (III)
11.	UBASK31	33.32 ± 19.93 a (IV)	46.63 ± 13.49 a (V)
12.	UBASK32	51.76 ± 19.19 a (VI)	52.23 ± 10.25 a (VI)
13.	UBASK34	46.55 ± 4.09 a (V)	48.87 ± 4.59 a (V)
14.	UBASK35	20.57 ± 13.31 a (II)	33.76 ± 10.79 b (IV)
15.	UBASK36	49.07 ± 29.08 a (V)	50.39 ± 12.75 a (V)
16.	UBASK41	33.95 ± 8.88 a (IV)	42.40 ± 10.24 a (V)
17.	UBASK42	27.75 ± 6.80 a (III)	39.36 ± 9.05 b (IV)
18.	UBASK43	25.16 ± 12.07 a (III)	43.02 ± 1.64 a (V)
19.	UBASK45	41.13 ± 14.72 a (V)	27.96 ± 3.83 b (III)
20.	UBASK46	40.71 ± 10.98 a (IV)	46.56 ± 13.45 a (V)
21.	UBASK51	27.46 ± 14.82 a (III)	29.36 ± 12.00 b (III)
22.	UBASK52	41.05 ± 19.21 a (V)	38.18 ± 22.40 b (IV)
23.	UBASK53	45.07 ± 24.79 a (V)	24.02 ± 1.22 b (III)
24.	UBASK54	31.31 ± 15.80 a (IV)	24.07 ± 1.74 b (III)
25.	UBASK56	31.39 ± 18.56 a (IV)	29.58 ± 10.72 b (III)
26.	UBASK61	32.98 ± 18.65 a (IV)	49.57 ± 10.80 a (V)
27.	UBASK62	48.43 ± 22.58 a (V)	61.20 ± 11.39 a (VII)
28.	UBASK63	35.75 ± 14.01 a (IV)	38.75 ± 13.10 b (IV)
29.	UBASK64	47.61 ± 8.34 a (V)	59.85 ± 29.19 a (VI)
30.	UBASK65	42.40 ± 16.23 a (V)	32.32 ± 8.72 b (IV)
31.	AGP	38.21 ± 19.87 a (IV)	38.93 ± 22.18 b
32.	AJM	37.52 ± 4.29 a (IV)	23.46 ± 3.60 b
33.	GBG	22.79 ± 6.37 a (III)	30.15 ± 9.31 b
34.	TGM	26.79 ± 22.83 a (III)	23.39 ± 7.36 b
35.	UB 1	32.81 ± 16.31 a (IV)	28.44 ± 9.29 b
36.	UB 2	13.16 ± 10.83 a (II)	18.31 ± 10.64 b

**Note:**

Pest damage severity was assessed using a modified visual rating scale ranging from I–X: I = 0–10%, II = >10–20%, III = >20–30%, IV = >30–40%, V = >40–50%, VI = >50–60%, VII = >60–70%, VIII = >70–80%, IX = >80–90%, and X = >90–100% leaf damage (Hanafiah, 2010). Values are presented as mean ± standard deviation (SD). Different letters within the same column indicate statistically distinct groups based on the Scott–Knott test at the 5% significance level (a = higher intensity; b = lower intensity). Numbers in parentheses represent visual damage severity classes.

At 41 DAP, differences among genotypes became clearer, with the Scott–Knott test separating soybean lines into two distinct groups. The higher-intensity group (a) included genotypes such as UBASK62 (61.20 ± 11.39), UBASK64 (59.85 ± 29.19), UBASK15 (57.70 ± 11.03), UBASK14 (56.58 ± 9.64), UBASK32 (52.23 ± 10.25), UBASK36 (50.39 ± 12.75), UBASK61 (49.57 ± 10.80), and UBASK34 (48.87 ± 4.59). These genotypes consistently exhibited higher leaf injury, indicating stronger susceptibility to armyworm feeding at later vegetative stages.

In contrast, the lower-intensity group (b) comprised genotypes with substantially reduced attack levels. The most resistant entries included UB 2 (18.31 ± 10.64), UBASK24 (19.43 ± 1.29), AJM (23.46 ± 3.60), TGM (23.39 ± 7.36), UBASK53 (24.02 ± 1.22), UBASK54 (24.07 ± 1.74), UBASK25 (25.96 ± 5.25), UBASK26 (27.05 ± 14.06), UB 1 (28.44 ± 9.29), and UBASK51 (29.36 ± 12.00) (Table 1). These genotypes performed consistently better, maintaining lower intensities of feeding damage and reflecting stronger expression of resistance traits.

Several genotypes transitioned from moderate numerical intensity at 36 DAP to clearly lower intensity at 41 DAP for example GBG (30.15 ± 9.31), UBASK21 (30.78 ± 15.66), UBASK23 (30.93 ± 6.27), and UBASK63 (38.75 ± 13.10). This indicates that resistance expression strengthened as plants aged. Conversely, genotypes such as UBASK14, UBASK32, UBASK62, and UBASK64 consistently remained in the higher-intensity (a) category, marking them as more susceptible across both stages of observation.

### Average frequency of armyworm attacks on soybean genotypes

The frequency of *Spodoptera frugiperda* attacks at 36 days after planting (DAP) varied significantly among soybean genotypes, forming clear high- and low-frequency groups based on the Scott–Knott classification. Genotypes belonging to the higher-frequency group included UBASK16 (87.04 ± 11.56), UBASK14 (83.15 ± 15.72), UBASK15 (91.11 ± 15.40) (Table 2), and several other genotypes positioned within the medium to high attack range. These entries experienced more frequent larval feeding and thus demonstrated higher susceptibility at this early growth stage. In contrast, the lower-frequency group consisted of genotypes such as UB 2 (26.41 ± 18.62), UBASK42 (47.22 ± 3.06), and TGM (42.14 ± 23.20) (Table 2). These genotypes displayed substantially reduced pest visitations and foliar feeding, indicating stronger early-stage resistance. UB 2 exhibited the lowest attack frequency among all entries at 36 DAP.

**Table 2:** Average frequency (%) of armyworm (*Spodoptera frugiperda*) attacks on 36 soybean genotypes at 36 and 41 days after planting (DAP).

No.	Genotype	Non-selected (%)	
		36 DAP	41 DAP
1.	UBASK12	66.49 ± 20.33 a (M)	74.76 ± 5.36 a (M)
2.	UBASK13	59.66 ± 23.27 b (M)	66.45 ± 9.94 a (M)
3.	UBASK14	83.15 ± 15.72 a (H)	92.08 ± 9.71 a (H)
4.	UBASK15	91.11 ± 15.40 a (H)	78.32 ± 9.74 a (H)
5.	UBASK16	87.04 ± 11.56 a (H)	77.03 ± 9.88 a (H)
6.	UBASK21	60.82 ± 25.24 b (M)	49.43 ± 14.06 b (L)
7.	UBASK23	67.70 ± 9.95 a (M)	62.23 ± 8.28 b (M)
8.	UBASK24	44.92 ± 13.33 b (L)	50.20 ± 12.20 b (L)
9.	UBASK25	56.33 ± 15.04 b (M)	39.09 ± 16.93 b (L)
10.	UBASK26	55.67 ± 15.79 b (M)	45.48 ± 15.54 b (L)
11.	UBASK31	63.43 ± 22.32 b (M)	82.28 ± 9.73 a (H)
12.	UBASK32	81.23 ± 15.68 a (H)	85.14 ± 4.51 a (H)
13.	UBASK34	84.24 ± 14.12 a (H)	95.49 ± 5.35 a (H)
14.	UBASK35	42.29 ± 27.66 b (L)	73.50 ± 4.53 a (M)
15.	UBASK36	74.62 ± 14.76 a (M)	79.90 ± 9.11 a (H)
16.	UBASK41	67.43 ± 20.28 a (M)	79.83 ± 11.92 a (H)
17.	UBASK42	47.22 ± 3.06 b (L)	51.12 ± 3.27 b (M)
18.	UBASK43	54.60 ± 25.55 b (M)	72.15 ± 8.96 a (M)
19.	UBASK45	68.06 ± 12.92 a (M)	51.26 ± 6.62 b (M)
20.	UBASK46	78.34 ± 9.39 a (H)	81.08 ± 5.63 a (H)
21.	UBASK51	55.82 ± 18.16 b (M)	52.81 ± 17.63 b (M)
22.	UBASK52	67.32 ± 14.35 a (M)	58.38 ± 24.02 b (M)
23.	UBASK53	67.88 ± 28.26 a (M)	45.06 ± 4.19 b (L)
24.	UBASK54	56.78 ± 14.79 b (M)	46.25 ± 7.21 b (L)
25.	UBASK56	59.02 ± 19.16 b (M)	48.85 ± 8.23 b (L)
26.	UBASK61	71.07 ± 20.25 a (M)	88.29 ± 16.80 a (H)
27.	UBASK62	84.34 ± 17.45 a (H)	88.27 ± 8.17 a (H)
28.	UBASK63	70.57 ± 1.40 a (M)	78.89 ± 10.72 a (H)
29.	UBASK64	86.45 ± 19.67 a (H)	88.32 ± 11.29 a (H)
30.	UBASK65	77.01 ± 14.52 a (H)	72.72 ± 15.49 a (M)
31.	AGP	56.24 ± 24.58 b (M)	56.06 ± 13.87 b (M)
32.	AJM	67.58 ± 6.50 a (M)	53.54 ± 11.80 b (M)
33.	GBG	45.42 ± 3.32 b (L)	58.46 ± 6.91 b (M)
34.	TGM	42.14 ± 23.20 b (L)	42.61 ± 9.80 b (L)
35.	UB 1	62.87 ± 25.25 b (M)	43.25 ± 6.11 b (L)
36.	UB 2	26.41 ± 18.62 b (L)	33.38 ± 16.92 b (L)

**Note:**

Values represent the percentage of feeding frequency expressed as mean ± standard deviation (SD). Different letters within the same column indicate statistically distinct groups based on the Scott–Knott test at the 5% probability level (a = higher frequency, b = lower frequency). Letters within parentheses indicate qualitative severity classes: L = Low (<50% leaves attacked), M = Medium (50–75% leaves attacked), H = High (>75% leaves attacked) (Syahrawati & Busniah, 2009).

At 41 DAP, statistically significant differences persisted, and the high- and low-frequency groupings remained consistent with the earlier trend. Genotypes forming the higher-frequency group included UBASK14 (92.08 ± 9.71), UBASK34 (95.49 ± 5.35), UBASK16 (77.03 ± 9.88) (Table 2), and several additional genotypes that maintained elevated pest attack frequency. These genotypes consistently demonstrated susceptibility under increasing pest pressure. Conversely, the lower-frequency group included UB 2 (33.38 ± 16.92), UBASK25 (39.09 ± 16.93), TGM (42.61 ± 9.80), UB 1 (43.25 ± 6.11), UBASK21 (49.43 ± 14.06), UBASK24 (50.20 ± 12.20), and UBASK42 (51.12 ± 3.27) (Table 2). These genotypes consistently showed low attack frequencies across both time points, indicating stable resistance expression and reduced larval feeding preference.

A general decline in attack frequency from 36 to 41 DAP was observed, which could be attributed to larval mortality and reduced feeding activity in late instar larvae. Additionally, rainfall during the study period (352.5 mm at February, 348.7 mm at March, 354.6 at April, and 74.6 mm at May; BPS, 2022) may have contributed to larval mortality, aligning with findings by Nagoshi et al. (2023), which reported that intense and frequent rainfall negatively impacts *S. frugiperda* survival.

### Classification of soybean genotypes based on resistance to *Spodoptera frugiperda*

The resistance of soybean genotypes to *Spodoptera frugiperda* was categorized based on the method proposed by Chiang & Talekar (1980), which considers the standard deviation of attack intensity across tested genotypes (Table 3). The categorization results revealed variations in resistance levels, with some genotypes exhibiting high susceptibility (HS), while others demonstrated resistant (R) or strong resistance (SR). Among the 36 tested genotypes, UB 2 displayed the highest resistance (*SR*), while UBASK35, UBASK54, TGM, and GBG were classified as resistant (*R*). Conversely, UBASK15, UBASK62, UBASK64, UBASK32, and UBASK36 were identified as highly susceptible (*HS*), indicating their vulnerability to *S. frugiperda* infestation.

**Table 3:** Classification of 36 soybean genotypes based on average attack intensity of *Spodoptera frugiperda*.

No.	Genotype	Non-selected (%)	
		Average attack intensity	Category
1.	UBASK12	38.15 ± 5.43	S
2.	UBASK13	30.70 ± 2.57	MR
3.	UBASK14	48.16 ± 11.91	HS
4.	UBASK15	56.20 ± 2.12	HS
5.	UBASK16	42.48 ± 7.69	S
6.	UBASK21	30.80 ± 0.03	MR
7.	UBASK23	40.29 ± 13.23	S
8.	UBASK24	24.76 ± 7.54	R
9.	UBASK25	34.45 ± 12.00	MR
10.	UBASK26	32.33 ± 7.47	MR
11.	UBASK31	39.98 ± 9.41	S
12.	UBASK32	51.99 ± 0.34	HS
13.	UBASK34	47.71 ± 1.64	HS
14.	UBASK35	27.16 ± 9.33	R
15.	UBASK36	49.73 ± 0.94	HS
16.	UBASK41	38.18 ± 5.98	S
17.	UBASK42	33.56 ± 8.21	MR
18.	UBASK43	34.09 ± 12.63	MR
19.	UBASK45	34.55 ± 9.32	MR
20.	UBASK46	43.63 ± 4.14	S
21.	UBASK51	28.41 ± 1.34	MR
22.	UBASK52	39.62 ± 2.03	S
23.	UBASK53	34.55 ± 14.89	MR
24.	UBASK54	27.69 ± 5.12	R
25.	UBASK56	30.49 ± 1.28	MR
26.	UBASK61	41.28 ± 11.73	S
27.	UBASK62	54.82 ± 9.03	HS
28.	UBASK63	37.25 ± 2.12	S
29.	UBASK64	53.73 ± 8.65	HS
30.	UBASK65	37.36 ± 7.13	S
31.	AGP	38.57 ± 0.51	S
32.	AJM	30.49 ± 9.94	MR
33.	GBG	26.47 ± 5.21	R
34.	TGM	25.09 ± 2.40	R
35.	UB 1	30.62 ± 3.09	MR
36.	UB 2	15.73 ± 3.65	SR

**Note:**

Average attack intensity values are presented as mean ± standard deviation (SD). Genotypes were categorized into five resistance classes based on their mean damage intensity using the Scott–Knott clustering test at the 5% significance level. Categories include: HS, Highly Susceptible; S, Susceptible; MR, Moderately Resistant; R, Resistant; and SR, Strongly Resistant (Chiang & Talekar, 1980).

### Growth performance of soybean genotypes under *Spodoptera frugiperda* infestation

Morphophysiological traits among the soybean genotypes exhibited substantial variation based on Scott–Knott grouping. Several lines showed higher leaf trichome density, including UBASK14, UBASK24, UBASK26, UBASK35, UBASK42, UBASK45, UBASK51, UBASK52, UBASK53, UBASK54, UBASK56, UBASK61, UBASK62, and GBG (Table 4), reflecting potentially stronger physical defense structures, whereas the remaining genotypes expressed lower LTD values. Leaf area at 4 and 6 DAP displayed no statistical differences, with all genotypes grouped in a, indicating uniform early vegetative development (Table 4).

**Table 4:** Morphophysiological characteristics of 36 soybean genotypes evaluated under armyworm (*Spodoptera frugiperda*) infestation.

No.	Genotype	LTD (0.25 cm^−2^)	LA 4 DAP (cm^2^)	LA 6 DAP (cm^2^)	Plant height (cm)	Number of leaf (trifoliate)	Time of flowering (DAP)
1.	UBASK12	20.89 ± 1.71 b	90.89 ± 21.17 a	75.25 ± 17.81 a	67.83 ± 9.39 a	13.67 ± 1.04 a	44.33 ± 2.89 a
2.	UBASK13	19.67 ± 4.26 b	95.42 ± 7.46 a	84.85 ± 27.86 a	56.83 ± 2.40 b	15.67 ± 1.26 a	42.67 ± 2.89 a
3.	UBASK14	40.89 ± 5.93 a	95.14 ± 11.44 a	92.61 ± 8.36 a	56.00 ± 1.89 b	11.00 ± 0.87 b	39.33 ± 2.89 b
4.	UBASK15	26.33 ± 4.81 b	80.81 ± 15.96 a	50.82 ± 2.87 a	56.17 ± 17.85 b	15.67 ± 4.48 a	37.00 ± 3.46 c
5.	UBASK16	23.56 ± 7.82 b	70.64 ± 6.81 a	65.88 ± 29.42 a	71.25 ± 10.89 a	11.83 ± 1.04 b	44.33 ± 2.89 a
6.	UBASK21	21.00 ± 6.01 b	86.66 ± 24.90 a	83.18 ± 2.86 a	63.50 ± 7.86 a	9.67 ± 1.04 b	41.00 ± 0.00 b
7.	UBASK23	18.56 ± 3.10 b	113.61 ± 13.27 a	93.40 ± 9.61 a	58.00 ± 4.77 b	10.00 ± 1.32 b	41.00 ± 0.00 b
8.	UBASK24	30.67 ± 4.04 a	97.50 ± 32.50 a	86.85 ± 14.09 a	74.33 ± 5.39 a	13.33 ± 1.53 a	41.00 ± 0.00 b
9.	UBASK25	27.67 ± 12.34 b	98.57 ± 10.38 a	84.13 ± 28.05 a	70.83 ± 2.25 a	13.67 ± 0.76 a	41.00 ± 0.00 b
10.	UBASK26	33.00 ± 14.53 a	93.08 ± 29.75 a	90.46 ± 15.43 a	63.83 ± 7.23 a	15.67 ± 6.05 a	41.00 ± 0.00 b
11.	UBASK31	23.11 ± 4.44 b	99.38 ± 14.70 a	65.14 ± 4.59 a	46.92 ± 13.39 c	12.50 ± 3.28 b	39.33 ± 2.89 b
12.	UBASK32	24.33 ± 3.79 b	103.39 ± 3.95 a	70.44 ± 7.12 a	68.50 ± 3.61 a	10.33 ± 0.29 b	41.00 ± 0.00 b
13.	UBASK34	23.22 ± 2.34 b	93.47 ± 5.08 a	86.52 ± 11.98 a	53.25 ± 7.38 b	11.50 ± 3.12 b	37.67 ± 2.89 c
14.	UBASK35	29.78 ± 9.76 a	100.16 ± 22.55 a	66.96 ± 9.68 a	67.75 ± 6.55 a	13.33 ± 1.89 a	42.67 ± 2.89 a
15.	UBASK36	20.44 ± 3.20 b	93.71 ± 9.44 a	64.87 ± 14.42 a	47.17 ± 12.64 c	14.50 ± 1.73 a	44.33 ± 2.89 a
16.	UBASK41	27.67 ± 6.36 b	91.80 ± 31.11 a	72.81 ± 26.30 a	71.92 ± 6.03 a	13.50 ± 2.29 a	41.00 ± 0.00 b
17.	UBASK42	42.11 ± 9.43 a	106.41 ± 31.66 a	106.43 ± 22.00 a	46.00 ± 3.50 c	11.67 ± 0.58 b	41.00 ± 0.00 b
18.	UBASK43	19.78 ± 5.64 b	78.13 ± 7.78 a	49.55 ± 22.76 a	59.92 ± 2.36 b	12.00 ± 2.00 b	42.67 ± 2.89 a
19.	UBASK45	30.11 ± 14.58 a	93.16 ± 13.38 a	73.39 ± 39.25 a	67.00 ± 5.27 a	12.33 ± 0.58 b	43.00 ± 0.00 a
20.	UBASK46	23.22 ± 8.77 b	95.11 ± 8.00 a	80.47 ± 9.54 a	49.67 ± 3.96 c	12.83 ± 2.02 b	41.00 ± 0.00 b
21.	UBASK51	40.11 ± 0.69 a	113.82 ± 15.09 a	93.55 ± 21.48 a	61.00 ± 0.87 a	12.00 ± 0.87 b	41.00 ± 0.00 b
22.	UBASK52	31.33 ± 12.29 a	64.96 ± 17.60 a	51.64 ± 13.04 a	66.17 ± 6.66 a	12.67 ± 2.57 b	41.00 ± 0.00 b
23.	UBASK53	28.89 ± 20.63 a	90.67 ± 3.74 a	66.72 ± 20.78 a	64.50 ± 5.77 a	15.50 ± 1.32 a	41.00 ± 0.00 b
24.	UBASK54	35.56 ± 1.35 a	99.58 ± 21.19 a	79.89 ± 16.06 a	68.83 ± 3.33 a	13.00 ± 0.50 b	35.33 ± 0.58 c
25.	UBASK56	37.44 ± 12.15 a	93.96 ± 23.01 a	90.69 ± 13.74 a	67.33 ± 0.58 a	13.50 ± 1.80 a	41.00 ± 0.00 b
26.	UBASK61	32.22 ± 4.44 a	119.70 ± 20.00 a	89.73 ± 22.84 a	78.67 ± 5.25 a	11.33 ± 1.76 b	41.00 ± 0.00 b
27.	UBASK62	28.22 ± 7.04 a	108.91 ± 14.19 a	78.51 ± 24.44 a	66.83 ± 11.25 a	14.83 ± 3.06 a	42.67 ± 2.89 a
28.	UBASK63	13.56 ± 4.35 b	91.98 ± 6.38 a	71.38 ± 44.76 a	51.25 ± 3.44 c	12.17 ± 1.26 b	39.00 ± 3.46 b
29.	UBASK64	37.11 ± 1.02 a	88.82 ± 16.11 a	80.04 ± 30.33 a	56.42 ± 8.30 b	14.00 ± 1.80 a	44.33 ± 2.89 a
30.	UBASK65	17.67 ± 4.70 b	101.24 ± 13.62 a	74.65 ± 19.99 a	38.42 ± 1.59 c	13.33 ± 1.26 a	35.67 ± 0.58 c
31.	AGP	18.78 ± 5.93 b	74.91 ± 36.27 a	71.39 ± 29.44 a	49.67 ± 15.07 c	11.67 ± 2.75 b	37.00 ± 3.46 c
32.	AJM	26.56 ± 13.18 b	97.28 ± 16.59 a	63.05 ± 4.75 a	73.83 ± 2.02 a	12.17 ± 2.25 b	41.00 ± 0.00 b
33.	GBG	37.78 ± 2.67 a	108.90 ± 9.14 a	78.96 ± 40.84 a	59.50 ± 3.50 b	14.17 ± 1.26 a	31.00 ± 0.00 d
34.	TGM	18.67 ± 6.39 b	76.77 ± 5.30 a	48.50 ± 7.50 a	61.33 ± 2.75 a	16.17 ± 3.75 a	47.00 ± 0.00 a
35.	UB 1	32.00 ± 3.48 a	96.44 ± 38.23 a	71.56 ± 27.94 a	63.83 ± 7.65 a	11.67 ± 2.57 b	41.00 ± 0.00 b
36.	UB 2	34.89 ± 6.48 a	73.73 ± 33.52 a	74.18 ± 17.06 a	71.00 ± 8.05 a	16.50 ± 3.91 a	41.00 ± 0.00 b

**Note:**

Values represent mean ± standard deviation (SD) for leaf trichome density (LTD), leaf area (LA) at 4 and 6 DAP, plant height (PH), number of trifoliate leaves, and flowering time. LTD was measured per 0.25 cm^2^, and leaf area was quantified using digital image analysis. Different letters within each column indicate statistically distinct groups based on the Scott–Knott test at the 5% significance level (a = higher values; b–d = lower values).

Plant height showed wider differentiation, with UBASK12, UBASK16, UBASK24, UBASK31, UBASK36, and UBASK41 among the taller genotypes, while UBASK31, UBASK36, UBASK42, UBASK46, UBASK63, UBASK65, AGP, and others formed the shorter-height groups (Table 4). Number of trifoliate leaves ranged from higher values in genotypes such as UBASK12, UBASK13, UBASK15, UBASK24, UBASK25, UBASK26, UBASK35, UBASK36, UBASK41, UBASK53, UBASK56, UBASK62, UBASK64, TGM, UB 2 to lower-leaf genotypes including UBASK14, UBASK16, UBASK21, UBASK23, UBASK31, UBASK32, UBASK34, UBASK42, UBASK43, UBASK46, UBASK51, UBASK52, GBG, and others (Table 4).

Time of flowering showed the clearest separation, with earliest flowering occurring in UBASK15, UBASK34, UBASK54, GBG, AGP, and UBASK65, whereas latest flowering was observed in UBASK12, UBASK13, UBASK15, UBASK24, UBASK35, UBASK36, UBASK45, UBASK52, UBASK53, UBASK62, UBASK64, TGM, and UB 2 (Table 4). Collectively, these results reveal diverse morphological responses across genotypes, indicating distinct growth patterns and defense-related traits that may contribute variably to resistance against *S. frugiperda*.

### Yield performance and reproductive traits of soybean genotypes

Yield-related traits showed substantial variation among the soybean genotypes, with clear statistical group separation across parameters. The number of productive branches (NPB) was relatively uniform, as all genotypes fell under group a (2.00–5.33 branches), indicating stable branching potential across the population. In contrast, the number of fertile nodes (NFN) showed a strong stratification, with the highest values observed in group a (28.50–31.33 nodes) such as UBASK26 and TGM, while most other genotypes clustered into group b (21.00–25.67), group c (13.33–20.50), and the lowest-performing genotypes such as GBG and UBASK43 in group d (13.33–14.83). Pod production (NP) and filled pods (NFP) displayed similar patterns, with TGM (NP: 85.00; NFP: 84.50) and UBASK35 (NP: 71.33; NFP: 71.17) categorized in group a, representing the most prolific pod-setting genotypes. Meanwhile, genotypes such as UBASK14, UBASK23, UBASK34, UBASK42, UBASK53, AGP, GBG, and UB 1 consistently placed in group d for NFP (25.33–38.83), reflecting lower reproductive output.

Harvest index (HI) also differentiated the genotypes sharply, with group a (0.63–0.77) including UBASK12, UBASK14, UBASK15, UBASK24, UBASK35, UBASK41, UBASK42, UBASK52, UBASK53, UBASK54, UBASK62, GBG, and AJM, indicating more efficient biomass-to-yield conversion. In comparison, group b (0.46–0.60) consisted of UBASK23, UBASK31, UBASK36, UBASK45, UBASK46, UBASK51, UBASK61, UBASK56, TGM, and UB 1. The 100-seed weight (W100) showed the widest range of variation, with the highest values in group a (19.18–19.75 g) represented by UBASK24 and GBG, followed by group b (15.12–16.73 g), group c (12.10–14.08 g), and group d as the lightest-seeded cluster (7.84–12.85 g) dominated by UBASK12, UBASK13, UBASK31, UBASK36, UBASK43, TGM, and UB 2. Overall, Table 5 demonstrates highly diverse agronomic performance, with TGM, UBASK35, UBASK24, and GBG showing superior yield components, while several UBASK lines with lower NFN, NFP, and W100 values fall within the lower-yielding groups.

**Table 5:** Yield-related traits of 36 soybean genotypes evaluated under *Spodoptera frugiperda* infestation.

No.	Genotype	HA (DAP)	NPB	NFN	NP	NFP	HI	W100
1.	UBASK12	84.00 ± 0.00 a	4.00 ± 0.50 a	22.17 ± 2.08 b	54.17 ± 2.84 b	54.00 ± 2.65 b	0.63 ± 0.05 a	10.49 ± 1.58 d
2.	UBASK13	84.00 ± 0.00 a	3.00 ± 0.87 a	24.00 ± 0.50 b	64.00 ± 2.60 b	63.00 ± 3.04 b	0.57 ± 0.17 b	10.68 ± 0.71 d
3.	UBASK14	84.00 ± 0.00 a	2.50 ± 0.00 a	16.33 ± 2.47 c	35.67 ± 2.52 d	35.33 ± 2.52 d	0.72 ± 0.07 a	12.85 ± 1.43 c
4.	UBASK15	84.00 ± 0.00 a	4.33 ± 0.76 a	22.50 ± 7.37 b	50.50 ± 14.34 c	48.33 ± 15.45 c	0.66 ± 0.08 a	12.19 ± 2.14 c
5.	UBASK16	84.00 ± 0.00 a	3.50 ± 1.32 a	21.17 ± 3.75 b	63.83 ± 15.70 b	62.33 ± 16.30 b	0.64 ± 0.03 a	12.19 ± 0.85 c
6.	UBASK21	84.00 ± 0.00 a	3.17 ± 1.89 a	18.50 ± 6.06 c	58.83 ± 20.70 b	58.83 ± 20.70 b	0.69 ± 0.04 a	11.34 ± 0.49 d
7.	UBASK23	84.00 ± 0.00 a	2.00 ± 0.50 a	15.17 ± 2.84 c	38.33 ± 5.35 d	37.33 ± 4.54 d	0.51 ± 0.09 b	15.12 ± 0.87 b
8.	UBASK24	84.00 ± 0.00 a	3.17 ± 0.29 a	16.67 ± 3.51 c	37.33 ± 7.97 d	36.50 ± 7.00 d	0.64 ± 0.01 a	19.18 ± 0.71 a
9.	UBASK25	84.00 ± 0.00 a	3.67 ± 0.29 a	16.67 ± 1.61 c	49.17 ± 3.82 c	48.83 ± 3.40 c	0.68 ± 0.06 a	13.23 ± 1.28 c
10.	UBASK26	84.00 ± 0.00 a	5.33 ± 2.36 a	28.50 ± 6.26 a	51.17 ± 6.51 c	50.33 ± 6.01 c	0.64 ± 0.05 a	12.57 ± 2.75 c
11.	UBASK31	84.00 ± 0.00 a	3.67 ± 0.29 a	18.17 ± 1.76 c	46.33 ± 5.35 c	45.67 ± 5.25 c	0.52 ± 0.13 b	16.73 ± 5.20 b
12.	UBASK32	84.00 ± 0.00 a	3.33 ± 1.26 a	19.67 ± 7.52 c	42.33 ± 12.17 c	41.33 ± 11.51 c	0.62 ± 0.08 b	14.08 ± 1.99 c
13.	UBASK34	84.00 ± 0.00 a	3.83 ± 1.26 a	15.83 ± 3.75 c	36.50 ± 6.06 d	35.17 ± 6.03 d	0.76 ± 0.03 a	13.61 ± 1.02 c
14.	UBASK35	84.00 ± 0.00 a	3.50 ± 0.50 a	22.50 ± 1.50 b	71.33 ± 6.90 a	71.17 ± 6.81 a	0.65 ± 0.05 a	10.11 ± 0.33 d
15.	UBASK36	84.00 ± 0.00 a	3.83 ± 0.29 a	22.83 ± 2.25 b	56.00 ± 6.24 b	55.17 ± 5.80 b	0.51 ± 0.05 b	7.84 ± 1.00 d
16.	UBASK41	84.00 ± 0.00 a	3.83 ± 1.04 a	17.67 ± 1.15 c	46.83 ± 4.07 c	45.33 ± 3.33 c	0.76 ± 0.03 a	14.08 ± 0.43 c
17.	UBASK42	84.00 ± 0.00 a	4.00 ± 0.87 a	18.17 ± 1.89 c	38.83 ± 2.31 d	38.50 ± 1.73 d	0.66 ± 0.04 a	13.23 ± 0.71 c
18.	UBASK43	84.00 ± 0.00 a	2.67 ± 1.04 a	14.83 ± 1.04 c	25.67 ± 2.08 d	25.33 ± 1.53 d	0.68 ± 0.01 a	11.15 ± 0.33 d
19.	UBASK45	84.00 ± 0.00 a	3.67 ± 0.29 a	21.00 ± 3.61 b	63.50 ± 1.32 b	62.00 ± 1.50 b	0.60 ± 0.04 b	10.30 ± 0.43 d
20.	UBASK46	84.00 ± 0.00 a	4.50 ± 1.32 a	20.33 ± 1.26 c	41.50 ± 3.00 d	41.00 ± 2.29 c	0.59 ± 0.04 b	12.29 ± 0.91 c
21.	UBASK51	84.00 ± 0.00 a	4.17 ± 1.26 a	21.33 ± 4.04 b	44.17 ± 4.93 c	50.00 ± 17.11 c	0.60 ± 0.19 b	15.21 ± 0.33 b
22.	UBASK52	84.00 ± 0.00 a	3.50 ± 1.32 a	18.00 ± 7.21 c	44.17 ± 14.09 c	43.00 ± 13.23 c	0.72 ± 0.01 a	13.32 ± 1.02 c
23.	UBASK53	84.00 ± 0.00 a	3.33 ± 0.29 a	15.83 ± 3.62 c	37.17 ± 11.37 d	36.17 ± 10.54 d	0.69 ± 0.07 a	13.99 ± 0.33 c
24.	UBASK54	84.00 ± 0.00 a	3.33 ± 1.04 a	15.83 ± 0.76 c	49.67 ± 3.21 c	48.33 ± 3.75 c	0.73 ± 0.03 a	16.73 ± 5.52 b
25.	UBASK56	84.00 ± 0.00 a	4.33 ± 1.26 a	29.17 ± 5.03 a	59.50 ± 12.68 b	58.83 ± 13.19 b	0.56 ± 0.09 b	8.69 ± 0.59 d
26.	UBASK61	84.00 ± 0.00 a	4.00 ± 0.50 a	21.83 ± 0.76 b	49.83 ± 6.60 c	49.33 ± 6.45 c	0.62 ± 0.06 b	11.34 ± 0.28 d
27.	UBASK62	84.00 ± 0.00 a	3.67 ± 0.76 a	19.33 ± 5.25 c	50.17 ± 7.65 c	49.00 ± 7.81 c	0.71 ± 0.04 a	11.81 ± 1.56 c
28.	UBASK63	84.00 ± 0.00 a	3.67 ± 1.15 a	18.50 ± 3.91 c	45.33 ± 5.75 c	44.67 ± 5.51 c	0.67 ± 0.02 a	10.02 ± 1.00 d
29.	UBASK64	84.00 ± 0.00 a	4.50 ± 0.50 a	20.50 ± 5.22 c	50.00 ± 11.50 c	49.33 ± 10.75 c	0.63 ± 0.06 a	12.10 ± 1.18 c
30.	UBASK65	84.00 ± 0.00 a	3.67 ± 1.26 a	14.83 ± 2.36 c	39.67 ± 2.31 d	38.83 ± 2.93 d	0.73 ± 0.04 a	11.43 ± 0.16 d
31.	AGP	84.00 ± 0.00 a	3.00 ± 0.50 a	15.33 ± 5.48 c	28.00 ± 9.50 d	27.83 ± 9.50 d	0.65 ± 0.07 a	12.29 ± 1.93 c
32.	AJM	84.00 ± 0.00 a	3.17 ± 0.29 a	16.83 ± 3.88 c	51.33 ± 5.35 c	50.00 ± 6.38 c	0.66 ± 0.03 a	12.47 ± 0.28 c
33.	GBG	84.00 ± 0.00 a	2.33 ± 0.29 a	13.33 ± 2.02 c	28.67 ± 1.61 d	27.33 ± 0.76 d	0.77 ± 0.03 a	19.75 ± 1.28 a
34.	TGM	84.00 ± 0.00 a	5.33 ± 0.76 a	31.33 ± 2.08 a	85.00 ± 17.44 a	84.50 ± 17.80 a	0.46 ± 0.10 b	9.45 ± 0.43 d
35.	UB 1	84.00 ± 0.00 a	3.33 ± 0.29 a	17.00 ± 0.00 c	39.83 ± 5.58 d	38.67 ± 6.25 d	0.59 ± 0.20 b	12.85 ± 0.33 c
36.	UB 2	84.00 ± 0.00 a	4.17 ± 1.04 a	25.67 ± 2.47 b	49.17 ± 3.33 c	47.50 ± 2.65 c	0.64 ± 0.06 a	10.49 ± 0.49 d

**Note:**

Values represent mean ± standard deviation (SD) for number of productive branches (NPB), number of fertile nodes (NFN), total pods (NP), filled pods (NFP), harvest index (HI), and 100-seed weight (W100). Different letters within each column indicate statistically distinct groups based on the Scott–Knott test at the 5% significance level (a = higher values; b–d = lower values). Yield components were measured at physiological maturity to assess the impact of *S. frugiperda* infestation on reproductive performance among genotypes.

### Principal component analysis of soybean genotypes based on agro-physiological traits

**Figure 1:**
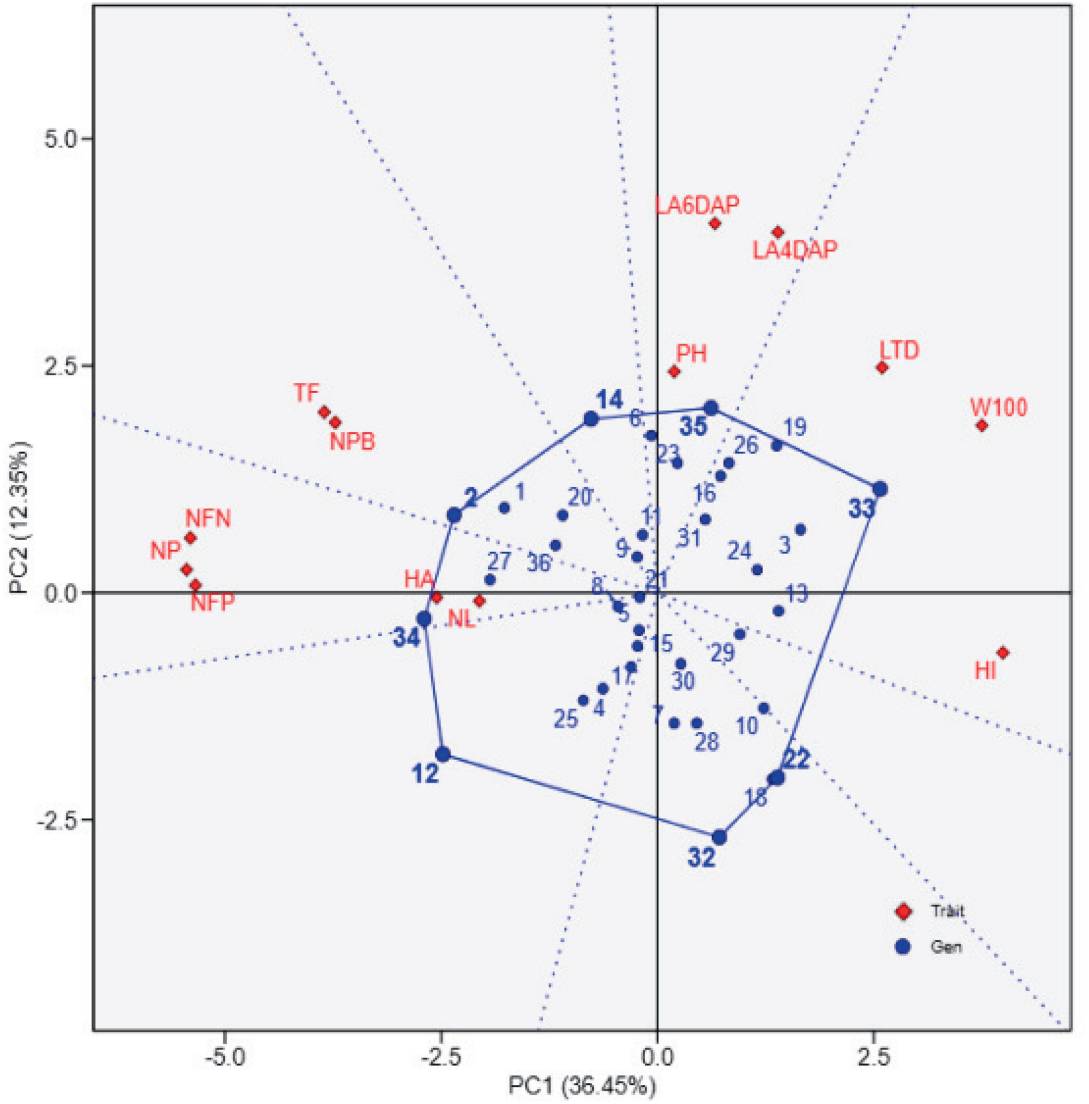
The biplot illustrates the relationships between soybean genotypes (blue points) and agrophysiological traits (red diamonds) based on principal component analysis (PCA). The first principal component (PC1) explains 36.45% of the total variation, while the second principal component (PC2) accounts for 12.35%. The polygon connects the outermost genotypes, highlighting those with extreme responses for specific traits. Red vectors represent the contribution and direction of traits, where longer vectors indicate stronger associations with the principal components. 1: UBASK16, 2: UBASK13, 3: UBASK14, 4: UBASK12, 5: UBASK15, 6: UBASK61, 7: UBASK63, 8: UBASK64, 9: UBASK62, 10: UBASK65, 11: UBASK31, 12: UBASK36 13: UBASK34, 14: UBASK32, 15: UBASK35,16: UBASK41, 17: UBASK46, 18: UBASK43, 19: UBASK42, 20: UBASK45, 21: UBASK21, 22: UBASK26, 23: UBASK23, 24: UBASK24, 25: UBASK25, 26: UBASK51, 27: UBASK56, 28: UBASK53, 29: UBASK54, 30: UBASK52, 31: AJM, 32: AGP, 33: GBG, 34: TGM, 35: UB 1, 36: UB 2.

The principal component analysis (PCA) biplot (Fig. 1) effectively differentiates soybean genotypes based on key agro-physiological traits, with PC1 (36.45% variance) capturing variability in yield-related traits such as the number of filled pods (NFP), 100-seed weight (W100), and harvest index (HI). The contrasting positions of NFP and W100/HI in the biplot likely reflect a trade-off between seed number and seed size, where genotypes with more filled pods tend to produce smaller seeds and lower harvest index due to resource allocation limits. Genotypes positioned positively along PC1, such as UBASK16 and UBASK13, exhibit superior reproductive attributes, making them potential candidates for high-yield selection. In contrast, genotypes on the negative side of PC1, including UBASK61 and UBASK63, show lower yield in comparison with high-performing genotypes, indicating a need for agronomic improvement. Meanwhile, PC2 (12.35% variance) primarily explains vegetative growth traits, such as leaf area (LA), number of leaves (NL), and plant height (PH), distinguishing genotypes with robust canopy development. However, excessive vegetative vigor without proportional reproductive success may result in inefficient resource partitioning, affecting seed production. The outermost genotypes form a polygon, highlighting those with extreme trait expressions, which could be valuable for targeted breeding strategies.

**Figure 2:**
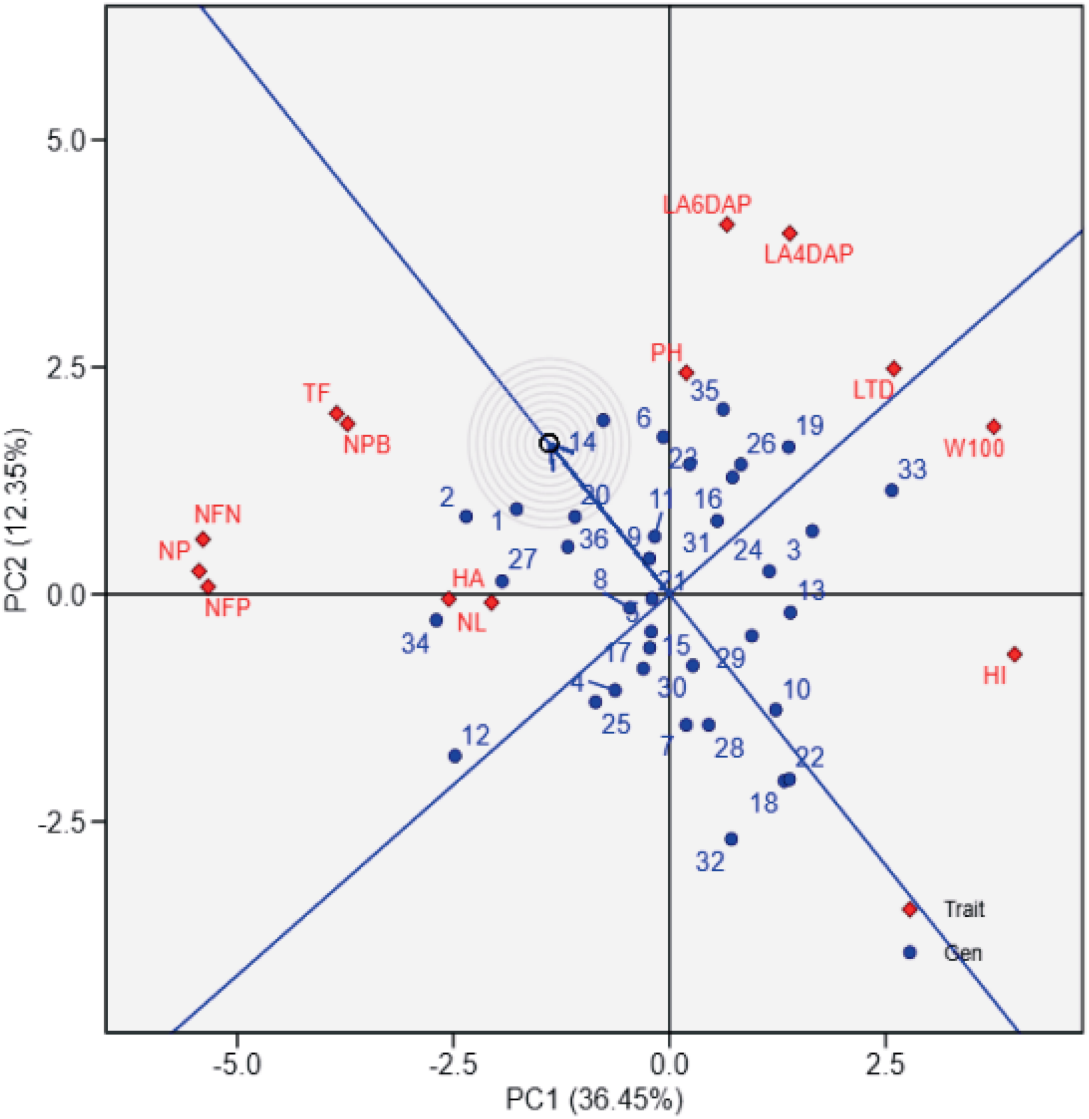
Ranking of soybean lines based on the ideal genotype in the genotype-by-trait biplot. The biplot shows the performance of soybean genotypes (blue points) relative to the ideal genotype (center of concentric circles) and agrophysiological traits (red diamonds) from principal component analysis (PCA). PC1 explains 36.45% of the variation, and PC2 explains 12.35%. The ideal genotype reflects balanced performance across traits, while the proximity of genotypes to this center aids in identifying high-performing lines. Red vectors indicate trait contributions, with longer vectors showing greater influence. 1: UBASK16, 2: UBASK13, 3: UBASK14, 4: UBASK12, 5: UBASK15, 6: UBASK61, 7: UBASK63, 8: UBASK64, 9: UBASK62, 10: UBASK65, 11: UBASK31, 12: UBASK36 13: UBASK34, 14: UBASK32, 15: UBASK35,16: UBASK41, 17: UBASK46, 18: UBASK43, 19: UBASK42, 20: UBASK45, 21: UBASK21, 22: UBASK26, 23: UBASK23, 24: UBASK24, 25: UBASK25, 26: UBASK51, 27: UBASK56, 28: UBASK53, 29: UBASK54, 30: UBASK52, 31: AJM, 32: AGP, 33: GBG, 34: TGM, 35: UB 1, 36: UB 2.

Figure 2 provides a ranking of soybean genotypes based on proximity to the ideal genotype, which represents an optimal balance across agronomic traits. UBASK16, UBASK14, and UBASK12 emerge as the closest to the ideal (middle) point, indicating their stability and potential for genetic improvement. Conversely, UBASK61 and UBASK42, which deviate significantly from the ideal genotype, may exhibit suboptimal performance across multiple traits. The contribution of individual traits is reflected in the vector lengths, where W100 and HI exert the greatest influence on PC1, signifying their importance in differentiating high-yielding lines. In contrast, NL and LA predominantly shape PC2, reinforcing the trade-off between vegetative and reproductive growth. These findings emphasize the necessity of balancing biomass accumulation with reproductive efficiency to optimize yield potential in soybean breeding programs.

### Interrelationship of agro-physiological traits based on path analysis

**Figure 3:**
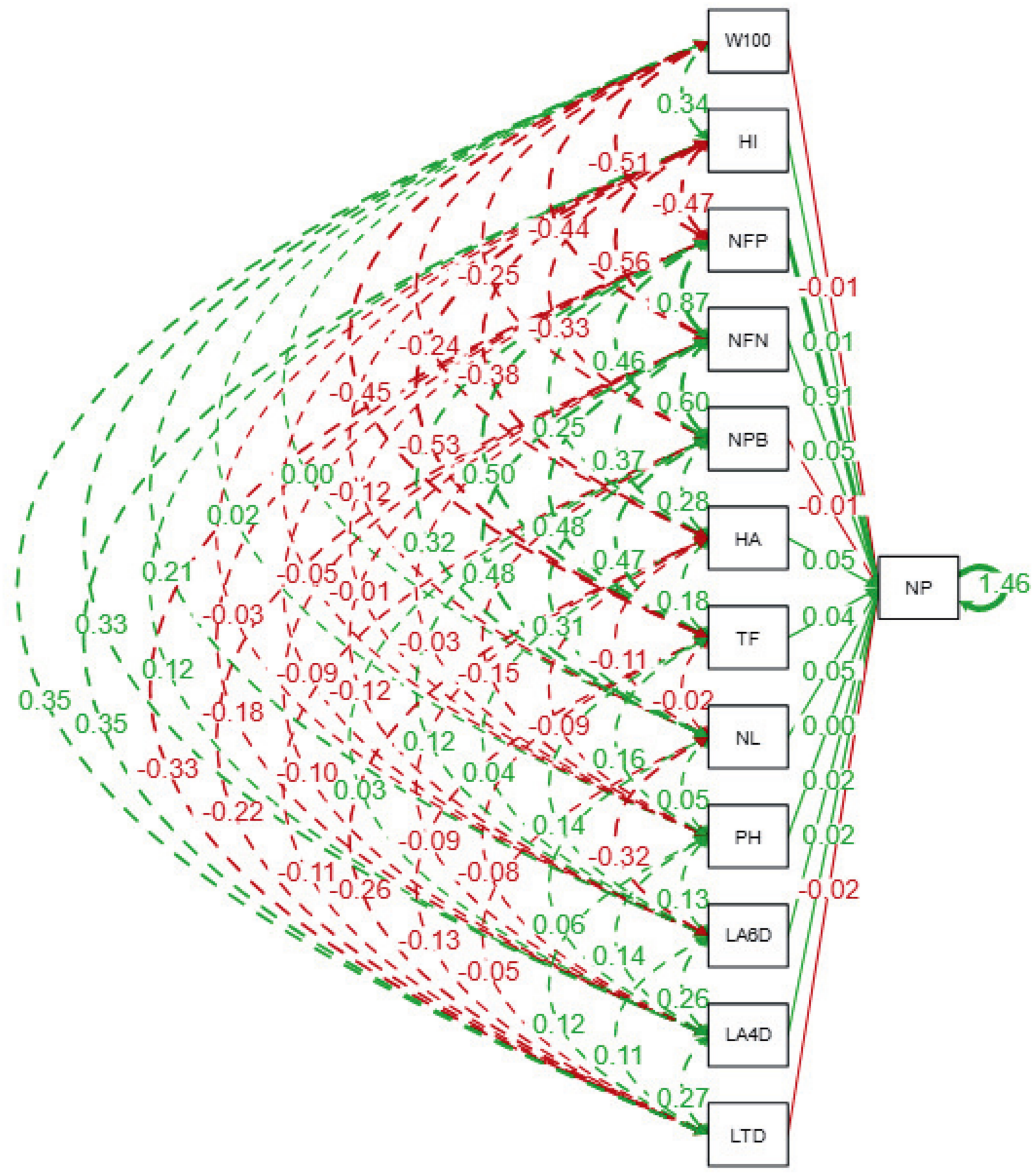
Path diagram showing direct and indirect relationships among agrophysiological traits of soybean lines. The diagram illustrates standardized path coefficients, where solid lines indicate direct effects, and dashed lines represent indirect effects among traits. Green lines represent positive relationships, while red lines indicate negative relationships. The thickness of the lines corresponds to the magnitude of the path coefficient. LTD, Leaf Trichome Density; LA, Leaf Area (DAP = Days After Planting); PH, Plant Height; NL, Number of Leaf; TF, Time of Flowering; HA, Harvest Age; NPB, Number of Productive Branches; NFN, Number of Fertile Nodes; NP, Number of Pods; NFP, Number of Filled Pods; HI, Harvest Index; W100, Weight of 100 Seeds.

The path analysis (Fig. 3 and Table 6) quantifies the direct and indirect effects of agro-physiological traits on the number of pods (NP) in soybean lines. The number of fertile nodes (NFN) exhibits the strongest direct positive effect on NP, with a standardized path coefficient of 0.89 (*p* ≤ 0.01). This highlights NFN as a critical trait for pod development, making it a primary focus for selection in breeding programs. Similarly, the number of productive branches (NPB) contributes significantly to NP with a direct effect of 0.49 (*p* ≤ 0.01), reinforcing its importance in enhancing pod formation. Traits such as time of flowering (TF) and harvest age (HA) also show moderate direct effects on NP, with coefficients of 0.52 and 0.29, respectively, indicating that earlier flowering and optimal harvest timing can positively influence pod production.

**Table 6:** Path analysis of the relationship between agrophysiological characteristics of soybean lines.

Traits	LTD	LA4DAP	LA6DAP	PH	NL	TF	HA	NPB	NFN	NP	NFP	HI	W100
LTD	1.00 NA	0.27	0.11	0.12	−0.05	−0.13	−0.26	−0.11	−0.22	−0.34*	−0.33*	0.35*	0.35*
LA4DAP	0.27	1.00 NA	0.26	0.14	0.06	−0.08	−0.09	0.03	−0.1	−0.15	−0.18	0.12	0.33*
LA6DAP	0.11	0.26	1.00 NA	0.13	−0.32	0.14	0.04	0.12	−0.12	−0.08	−0.09	−0.03	0.21
PH	0.12	0.14	0.13	1.00 NA	0.05	0.16	−0.09	−0.15	−0.03	0	−0.01	−0.05	0.02
NL	−0.05	0.06	−0.32	0.05	1.00 NA	−0.02	−0.11	0.31	0.48**	0.35*	0.32	−0.12	0
TF	−0.13	−0.08	0.14	0.16	−0.02	1.00 NA	0.18	0.47**	0.48**	0.52**	0.50**	−0.53**	−0.45**
HA	−0.26	−0.09	0.04	−0.09	−0.11	0.18	1.00 NA	0.28	0.37*	0.29	0.25	−0.38*	−0.24
NPB	−0.11	0.03	0.12	−0.15	0.31	0.47**	0.28	1.00 NA	0.60**	0.49**	0.46**	−0.33	−0.25
NFN	−0.22	−0.1	−0.12	−0.03	0.48**	0.48**	0.37*	0.60**	1.00 NA	0.89**	0.87**	−0.56**	−0.44**
NP	−0.34*	−0.15	−0.08	0	0.35*	0.52**	0.29	0.49**	0.89**	1.00 NA	0.99**	−0.48**	−0.51**
NFP	−0.33*	−0.18	−0.09	−0.01	0.32	0.50**	0.25	0.46**	0.87**	0.99**	1.00 NA	−0.47**	−0.51**
HI	0.35*	0.12	−0.03	−0.05	−0.12	−0.53**	−0.38*	−0.33	−0.56**	−0.48**	−0.47**	1.00 NA	0.34*
W100	0.35*	0.33*	0.21	0.02	0	−0.45**	−0.24	−0.25	−0.44**	−0.51**	−0.51**	0.34*	1.00 NA

**Note:**

LTD (Leaf Trichome Density), LA4DAP, LA6DAP (Leaf Area at 44 and 66 DAP), PH, Plant Height; NL, Number of Leaves; TF, Time of Flowering; HA, Harvest Age; NPB, Number of Productive Branches; NFN, Number of Fertile Nodes; NP, Number of Pods; NFP, Number of Filled Pods; HI, Harvest Index; and W100, 100-Seed Weight. Significance levels: **(*p* ≤ 0.01), *(*p* ≤ 0.05), (*p* ≤ 0.1). NA indicates non-significant paths. Diagonal values (1.00) show variable self-correlation.

Conversely, some traits exhibit negative direct effects on NP. The weight of 100 seeds (W100) and harvest index (HI) display significant negative coefficients of −0.51 and −0.48 (*p* ≤ 0.01), respectively, suggesting a trade-off between individual seed size, overall yield efficiency, and pod formation. Indirect contributions are also notable; for instance, leaf area at 4 days after planting (LA4DAP) and leaf area at 6 days after planting (LA6DAP) indirectly influence NP through their positive effects on NPB and NFN, despite their direct coefficients for NP being non-significant. Furthermore, leaf trichome density (LTD) has a significant negative direct effect on NP (−0.34, *p* ≤ 0.05), indicating its potential as a limiting factor for pod production, possibly due to reduced photosynthetic efficiency. The strongest indirect effects arise through the influence of NFN and NPB on NP, as evidenced by traits like number of leaves (NL) (indirect effect: 0.35, *p* ≤ 0.05) and time of flowering (TF) (indirect effect: 0.48, *p* ≤ 0.01). These findings emphasize the interconnected nature of vegetative and reproductive traits in determining soybean yield potential.

### Hierarchical cluster analysis of soybean lines based on agro-physiological traits and armyworm susceptibility

**Figure 4:**
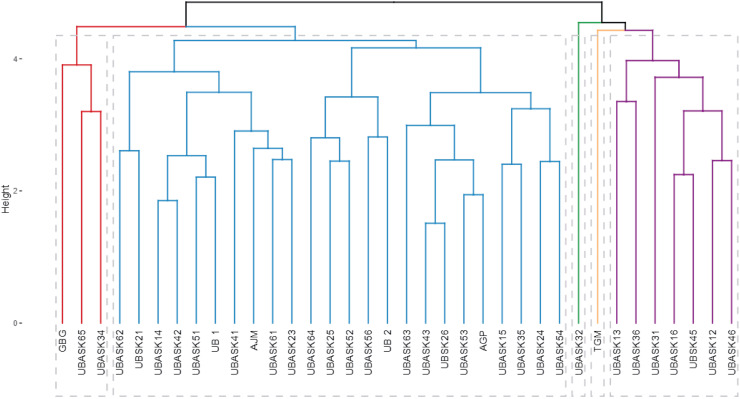
Hierarchical classification of soybean lines based on agrophysiological traits and susceptibility to armyworm attacks. The dendrogram shows clusters of soybean genotypes based on hierarchical cluster analysis (HCA), integrating agrophysiological characteristics, intensity, and frequency of armyworm attacks. The branch height represents dissimilarity, with shorter branches indicating higher similarity. Distinct clusters (colored branches) highlight genotypes with similar traits and susceptibility, aiding in the identification of resistant and high-performing lines for breeding purposes.

The dendrogram (Fig. 4) categorizes soybean genotypes into distinct clusters based on agro-physiological characteristics and their susceptibility to armyworm attacks, providing valuable insights for identifying resistant and high-performing lines. The hierarchical clustering process used branch height as a measure of dissimilarity, where shorter branches indicate greater similarity among genotypes. This analysis resulted in several clearly defined clusters, each representing genotypes with similar agro-physiological profiles and susceptibility levels. The clusters reveal patterns that are crucial for breeding purposes.

For instance, genotypes in Cluster Red are characterized by higher resistance to armyworm attacks, as evidenced by their lower intensity and frequency of infestation, coupled with favorable agro-physiological traits such as high number of productive branches (NPB) and number of fertile nodes (NFN). In contrast, genotypes in Cluster Blue exhibit greater susceptibility, reflected in higher infestation rates and less optimal agronomic traits, such as lower harvest index (HI) and reduced pod formation (NP). The identification of these resistant and susceptible clusters is instrumental for guiding the selection of parental lines in breeding programs aimed at enhancing pest resistance while maintaining or improving yield potential.

The dendrogram also highlights the potential trade-offs between resistance and agronomic performance in some clusters. For example, genotypes in Cluster Green demonstrate moderate resistance to armyworms but exhibit suboptimal values for certain yield-related traits, such as weight of 100 seeds (W100) and harvest index (HI). These genotypes could be targeted for improvement through breeding strategies that balance pest resistance with productivity. The distinct clustering patterns emphasize the utility of hierarchical cluster analysis (HCA) in integrating multiple traits, enabling comprehensive evaluations that align with both pest resistance and agronomic goals.

## Discussion

### Harnessing morphological defenses in soybean resistance to *Spodoptera frugiperda*

The efficacy of trichomes in mitigating *Spodoptera frugiperda* infestations is rooted in their intricate structural and biochemical properties that actively interfere with insect feeding behavior. From a mechanical perspective, high trichome density increases the leaf surface’s micro-topographical complexity, reducing the ability of larvae to navigate and secure stable attachment for feeding (Udayakumar, Shivalingaswamy & Bakthavatsalam, 2020). This disruption is particularly detrimental to *S. frugiperda*, as its feeding mechanism involves precise mandibular engagement with epidermal cells. When trichomes obstruct this interaction, larvae experience increased feeding difficulty, leading to prolonged foraging periods and reduced nutrient intake (Jin et al., 2020). Moreover, trichomes influence thermoregulation on the leaf surface, altering microclimatic conditions that deter insect settlement. In highly trichome-dense genotypes, localized temperature variations and reduced leaf wetness could further hinder larval survival, particularly during the early instar stages when moisture retention is crucial for cuticle hydration and molting (Thrash et al., 2013). These factors collectively explain why genotypes such as UBASK42, with dense trichome coverage, exhibited lower pest attack intensity and reduced feeding damage. The role of trichomes as a physical barrier has been widely documented across multiple insect species affecting soybean (Kaur et al., 2022). Consistent with these findings, the present study confirms that high trichome density also significantly hinders *Spodoptera frugiperda* larval feeding and survival.

Beyond physical deterrence, trichomes contribute to resistance through the biosynthesis and localized deposition of allelochemicals that disrupt insect physiology. Soybean trichomes are known to accumulate flavonoids, tannins, and terpenoid compounds that serve as secondary metabolites with defensive properties (Lytra et al., 2024). These compounds interfere with *S. frugiperda*’s digestive enzymes, particularly trypsin-like proteases and amylases, which are essential for breaking down plant macromolecules into absorbable nutrients (Boiça Júnior et al., 2016). The inhibition of these enzymes results in suboptimal digestion and assimilation of leaf tissue, leading to reduced larval growth rates and, in severe cases, increased mortality. Additionally, the biochemical composition of trichomes may induce oxidative stress in insects by generating reactive oxygen species (ROS) upon ingestion, damaging cellular structures within the midgut epithelium of *S. frugiperda* (Santos-Amaya et al., 2022). This mechanism not only reduces larval fitness but may also decrease fecundity in adult moths that emerge from affected larvae, potentially lowering future infestation rates. The alignment between these biochemical deterrents and reduced pest survival in trichome-rich soybean genotypes suggests that targeted selection for both structural and chemical resistance factors could enhance the durability of pest resistance in soybean breeding programs (Huang et al., 2023). Other biochemical defenses such as increased production of phenolic compounds, proteinase inhibitors, and flavonoids may also contribute to resistance. These compounds interfere with insect digestion, nutrient assimilation, or hormonal regulation, leading to delayed development or mortality (Santos-Alves et al., 2018). Pregulation of defensive secondary metabolites under pest pressure through jasmonic acid signaling may reinforces the multifaceted nature of soybean resistance.

Despite the benefits conferred by trichomes, their extensive biosynthesis imposes physiological trade-offs that may affect overall plant productivity. Trichome development is regulated by a network of transcription factors, including the MYB, bHLH, and WD-repeat (MBW) complexes, which also modulate pathways involved in reproductive growth and metabolic resource allocation (Gontijo et al., 2018). High trichome investment can lead to reduced availability of photosynthates for reproductive organs, delaying flowering and reducing seed set in some genotypes. Additionally, elevated trichome densities may alter stomatal function by influencing leaf boundary layer properties, potentially affecting transpiration efficiency under varying environmental conditions (Prasetyo & Hidayanto, 2016). From an agricultural perspective, these trade-offs necessitate a careful balance between enhancing trichome-mediated resistance and maintaining agronomic viability (Huang, 2020). While genotypes like UBASK42 demonstrate robust pest resistance, their potential productivity under different environmental conditions must be further evaluated to ensure that resistance traits do not inadvertently compromise yield stability.

### Genetic variability shapes resistance and agronomic performance in soybean genotypes

The genetic basis of resistance to *Spodoptera frugiperda* in soybean is a complex trait influenced by polygenic interactions that regulate morphological defenses, biochemical pathways, and physiological adaptability. Parental genotypes such as Argopuro and Tanggamus have contributed resistance alleles that enhance pest deterrence through trichome-mediated defense mechanisms and antibiosis factors (Maurer et al., 2024). The observed variation in resistance among genotypes is a direct consequence of differential gene expression patterns associated with trichome development, secondary metabolite production, and induced defense signaling. Studies on soybean resistance genetics suggest that key quantitative trait loci (QTLs), including GmT1 (*Glycine max* Trichome 1) and GmLOX2 (Lipoxygenase 2), are involved in regulating pest-induced responses (Houngbo et al., 2020). The differential inheritance of these loci among tested genotypes likely explains the clustering patterns observed in hierarchical clustering analysis (HCA), where resistant genotypes formed distinct genetic groups characterized by high trichome density, while susceptible genotypes lacked these protective features. This underscores the importance of parental lineage selection in breeding programs, as specific genotypic backgrounds confer superior resistance without compromising agronomic performance (Wang et al., 2025).

Beyond structural defenses, the variability in resistance mechanisms is also linked to inducible defense pathways governed by phytohormone signaling networks. Soybean genotypes with enhanced resistance, such as UBASK35 and UBASK54, exhibited strong activation of the jasmonic acid (JA) and salicylic acid (SA) pathways, which are critical in herbivore defense against *Spodoptera frugiperda* (Kanwal et al., 2024). The JA pathway mediates systemic responses by upregulating protease inhibitors, chitinases, and polyphenol oxidases, all of which interfere with *S. litura* digestion and development. Meanwhile, SA signaling enhances pathogenesis-related (PR) protein expression, contributing to localized cellular resistance (Chae et al., 2022). The variation in JA and SA pathway activation among soybean genotypes reflects underlying genetic divergence in hormonal regulation, which dictates whether plants prioritize defense over growth. This hormonal trade-off could explain why some genotypes exhibited high resistance but moderate yield reductions, as prolonged JA signaling can suppress auxin-mediated cell division, delaying reproductive development (Sotelo-Cardona et al., 2021). These findings emphasize that genetic screening for resistance should not be limited to structural traits alone but should incorporate molecular markers linked to phytohormonal efficiency, ensuring that resistance is balanced with growth potential.

The observed clustering of soybean genotypes in genotype-by-trait biplot analysis further reveals how genetic variability shapes agronomic performance under pest stress. Genotypes positioned closer to the “ideal genotype” centroid, such as UBASK35 and UBASK54, not only demonstrated strong resistance but also maintained high reproductive success, likely due to their optimized genetic resource allocation between defense and yield-related traits. Conversely, highly resistant but lower-yielding genotypes, such as UBASK42, were positioned further from the optimal zone, reflecting overinvestment in defense mechanisms at the expense of seed production. This pattern supports the evolutionary theory that plant resistance is often associated with fitness costs, where excessive resource allocation toward defense can reduce competitive ability in non-stress conditions (Ayala et al., 2024).

### The trade-off between pest resistance and yield stability in soybean cultivation

The inherent trade-off between pest resistance and yield stability in soybean arises from the plant’s metabolic resource distribution, where investment in defense mechanisms often comes at the expense of growth and reproductive efficiency (Gandham et al., 2024). Resistance traits such as trichome formation, secondary metabolite synthesis, and inducible defense pathways impose substantial energetic costs on the plant, requiring significant carbon and nitrogen allocation (Nagoshi et al., 2023). These resources, which would otherwise be directed toward cellular expansion, seed development, and nutrient assimilation, are instead diverted to defensive structures and chemical deterrents. This metabolic shift is particularly evident in highly resistant genotypes like UBASK42, which exhibit high trichome densities but suffer moderate reductions in seed yield. The metabolic cost hypothesis suggests that plants must optimize their energy use between survival and reproduction, explaining why soybean lines with constitutive resistance mechanisms may exhibit delayed pod filling, reduced seed weight, or altered nutrient partitioning under pest-free conditions (Tao et al., 2024).

Yield penalties in pest-resistant genotypes can also be attributed to the molecular crosstalk between stress response pathways and reproductive signaling networks. In highly resistant soybean genotypes, upregulation of defense-associated transcription factors (*e.g*., WRKY, MYC, and ERF families) can interfere with the hormonal balance required for reproductive phase transitions (Yifei et al., 2018). WRKY transcription factors, for instance, play a key role in activating systemic acquired resistance (SAR) but are also known to negatively regulate gibberellin biosynthesis, a hormone essential for stem elongation and floral induction (Saritha & Visalakshi, 2024). This antagonistic interaction results in delayed flowering, reduced pod-set efficiency, and smaller seed size in resistant genotypes. Additionally, excessive investment in defense-related lignification, which strengthens epidermal cell walls to deter *Spodoptera frugiperda*, may inadvertently reduce phloem loading efficiency, limiting the translocation of carbohydrates to developing seeds (Souza et al., 2021). This unintended physiological consequence highlights the challenge of maintaining high yield potential while ensuring robust pest resistance, necessitating breeding strategies that fine-tune gene expression without overcompensating for defense at the expense of productivity.

From an agroecological perspective, the trade-off between pest resistance and yield stability also has broader implications for cropping system resilience and long-term sustainability. In monoculture systems where pest pressure fluctuates seasonally, maintaining constitutive resistance traits may lead to inefficient resource utilization, particularly in seasons with low pest prevalence (Chowdary, Harika & Katlam, 2024). This phenomenon is exacerbated in low-input agricultural systems, where nutrient availability is already limited, making the additional energy cost of maintaining high trichome densities or secondary metabolite biosynthesis unsustainable. A potential solution lies in the development of semi-resistant or inducible resistance genotypes, which activate defense mechanisms only when pest attacks occur, thereby preserving resources for reproductive processes during non-stress conditions (Yadav & Singh, 2024). Additionally, integrating pest-resistant soybean cultivars into polyculture systems or precision pest management frameworks could further optimize their performance by reducing dependency on continuous resistance expression.

### Advancing sustainable agriculture through pest-resistant soybean genotypes

The development and integration of pest-resistant soybean genotypes play a pivotal role in promoting sustainable agricultural systems by reducing the reliance on synthetic pesticides while maintaining productivity. Conventional pest management strategies in soybean cultivation have historically depended on broad-spectrum chemical insecticides, which, while effective in the short term, contribute to pesticide resistance, biodiversity loss, and environmental contamination (Chattopadhyay et al., 2019). The excessive use of synthetic pesticides has led to the emergence of highly adaptive *Spodoptera frugiperda* populations, as evidenced by field reports of insecticide-resistant strains that exhibit increased detoxification enzyme activity, rendering many commercial insecticides ineffective. In contrast, genetically resistant soybean cultivars such as UBASK35 and UBASK54 provide a biologically inherent defense system, reducing the need for chemical inputs. By disrupting the pest’s feeding and reproductive cycles through trichome-mediated deterrence and secondary metabolite production, these genotypes contribute to long-term pest suppression, ultimately decreasing pesticide dependency and mitigating the negative environmental footprint of soybean production (Faiz et al., 2021).

Beyond direct pest resistance, the adoption of resistant soybean genotypes enhances agroecosystem resilience and soil health by minimizing pesticide-induced disruptions to non-target organisms, including pollinators, natural enemies of pests, and soil microbiota (Fattah et al., 2024). The widespread application of insecticides disrupts trophic interactions, leading to outbreaks of secondary pests due to the depletion of predatory arthropods such as *Coccinellidae* and *Chrysopidae*, which naturally regulate *S. frugiperda* populations (Hamawaki et al., 2012). Additionally, pesticide residues accumulate in the soil, altering microbial community structures essential for nutrient cycling, organic matter decomposition, and nitrogen fixation in legume-based cropping systems. By incorporating pest-resistant soybean varieties, farmers can maintain functional biodiversity within agricultural landscapes, ensuring that beneficial organisms continue to provide essential ecosystem services. This aligns with the principles of integrated pest management (IPM), which emphasize a multi-faceted approach to pest control that balances genetic resistance, biological control agents, and minimal chemical intervention to achieve long-term agricultural sustainability (Irshad et al., 2024).

The long-term success of pest-resistant soybean genotypes in sustainable agriculture depends on breeding advancements that enhance adaptability across diverse agroecological conditions. While current resistance traits such as high trichome density and secondary metabolite production are effective against *S. frugiperda*, the evolutionary adaptability of insect pests necessitates continuous genetic improvement to prevent resistance breakdown. One promising strategy is the incorporation of inducible resistance genes, which activate defense mechanisms only upon herbivore attack, reducing unnecessary metabolic costs during pest-free periods (Khan et al., 2022).

## Conclusions

This study revealed substantial genetic variation in soybean resistance to *Spodoptera frugiperda*. UB 2 was identified as the only strongly resistant (SR) genotype, while UBASK24, UBASK35, GBG, and TGM consistently formed the resistant (R) group, characterized by low damage levels and stable morpho-physiological performance. Path analysis showed that fertile nodes and productive branches were the strongest contributors to yield stability, reinforcing their value as selection criteria. Key resistance-related traits such as trichome density, flowering time, and yield components acted in combination rather than isolation, indicating a coordinated agro-physiological mechanism underlying resistance expression. Genotypes such as UBASK35 and UBASK54 demonstrated a favorable balance between resistance and productivity, underscoring their breeding potential. Overall, UB 2, UBASK24, UBASK35, GBG, and TGM represent strong candidates for developing soybean cultivars with improved resistance and agronomic resilience, although multi-environment validation remains essential to ensure their adaptation across diverse growing conditions.

Future research should include multi-season and multi-location trials to ensure the stability of resistance expression under varying environmental conditions. Genetic and genomic approaches such as QTL mapping, transcriptomic profiling, and CRISPR-based functional validation are recommended to identify key resistance genes and clarify the molecular basis of defense pathways. Integrating these resistant genotypes into long-term breeding pipelines and evaluating them within diverse integrated pest management (IPM) systems will be essential to optimize their performance and enhance sustainable soybean production.

## Supplemental Information

10.7717/peerj.20753/supp-1 Supplemental Information 1 Raw Data of Agrophysiological Traits in Soybean Genotypes for Resistance to Fall Armyworm. This dataset contains raw observations of agrophysiological traits in soybean genotypes. Variables include repetition, genotype, and measurements of leaf trichome density (across multiple leaves) and their averages. The data were collected to assess resistance to *Spodoptera litura* and are essential for analyzing the impact of morphological traits on pest resistance.

10.7717/peerj.20753/supp-2 Supplemental Information 2 Raw Data of Fall Armyworm Attack Intensity and Frequency in Soybean Genotypes. This dataset includes raw data on *Spodoptera litura* attack intensity and frequency in soybean genotypes. It provides information on the number of attacks, intensity scores, and frequency percentages, as well as the number of leaf samples analyzed. The data are critical for assessing the variation in pest resistance across genotypes under controlled field conditions.

10.7717/peerj.20753/supp-3 Supplemental Information 3 README: Documentation for Agrophysiological Traits in Soybean Genotypes. This README file provides detailed documentation for the dataset Raw Data of Agrophysiology Traits.csv. The dataset includes measurements of leaf trichome density across three leaves and their averages for various soybean genotypes. These observations were used to analyze the impact of morphological traits on resistance to *Spodoptera litura* and overall agronomic performance.

10.7717/peerj.20753/supp-4 Supplemental Information 4 Raw Data for Path Analysis and Dendrogram Clustering of Soybean Genotypes. This dataset provides raw data used for path analysis and dendrogram clustering of soybean genotypes. Variables include key agronomic traits such as leaf area at 4 and 6 days after planting (LA4DAP, LA6DAP), plant height (PH), number of leaves (NL), number of pods (NP), and 100-seed weight (W100). The data were collected to evaluate the interrelationships between traits contributing to resistance against *Spodoptera litura* and yield stability.

10.7717/peerj.20753/supp-5 Supplemental Information 5 R script used for ANOVA and Scott–Knott clustering analysis of soybean genotype data. This R script contains the complete workflow used to analyze soybean genotype performance under *Spodoptera frugiperda* infestation. The script performs data import, data reshaping into long format, analysis of variance (ANOVA), and subsequent mean separation using the Scott–Knott clustering method at the 5% significance level. The output includes the ANOVA summary, Scott–Knott grouping results, and a formatted table of genotype clusters.

10.7717/peerj.20753/supp-6 Supplemental Information 6 README: Documentation for Intensity and Frequency of Fall Armyworm Attacks in Soybean Genotypes. This README file provides comprehensive documentation for the dataset Raw Data of Intensity and Frequency.csv. The dataset includes data on the number of pest attacks, attack intensity, and frequency percentages observed in soybean genotypes under controlled field conditions. These variables were used to assess the resistance levels of genotypes to *Spodoptera litura* infestations.

10.7717/peerj.20753/supp-7 Supplemental Information 7 README: Documentation for Path and Dendrogram Analysis of Soybean Genotypes. This README file provides descriptive documentation for the dataset Raw Data of Path and Dendrogram Analysis.csv. It contains data on agronomic and physiological traits such as plant height, leaf area, number of pods, and 100-seed weight. These variables were analyzed to determine interrelationships among traits and to classify soybean genotypes based on their resistance to *Spodoptera litura* and yield stability.

## Additional Information and Declarations

### Data Availability

The following information was supplied regarding data availability:

The dataset is available at Zenodo: Karyawati, A. S. (2025). Raw Data for the Integrative Morphophysiological Assessment of Soybean Lines in Evaluating Leaf Rust Resistance [Data set]. Zenodo. https://doi.org/10.5281/zenodo.15081264.

## References

[ref-1] Ayala J, Vasquez A, Balakrishnan D, Madrigal E, George J, Kariyat R (2024). Effects of fast and slow-wilting soybean genotypes on fall armyworm (*Spodoptera frugiperda*) growth and development. Communicative & Integrative Biology.

[ref-2] Badan Pusat Statistik Kota Malang [BPS] (2022). Jumlah Curah Hujan di Kota Malang (milimeter (mm)). https://malangkota.bps.go.id/id/statistics-table/2/NTA4IzI=/jumlah-curah-hujan-di-kota-malang.html.

[ref-3] Boiça Júnior AL, Souza BH, Costa EN, Paiva LB (2016). Influence of fall armyworm previous experience with soybean genotypes on larval feeding behavior. Arthropod-Plant Interactions.

[ref-4] Chae H, Wen Z, Hootman T, Himes JG, Duan Q, McMath J, Ditillo JL, Sessler RJ, Conville J, Niu Y, Matthews P, Francischini FJ, Huang F, Bramlett MR (2022). *eCry1Gb.1Ig*, a novel chimeric cry protein with high efficacy against multiple fall armyworm (*Spodoptera frugiperda*) strains resistant to different GM traits. Toxins.

[ref-5] Chattopadhyay N, Balasubramaniam R, Attri SD, Ray K, John G, Khedikar S, Karmakar C (2019). Forewarning of incidence of *Spodoptera litura* (tobacco caterpillar) in soybean and cotton using statistical and synoptic approach. Journal of Agrometeorology.

[ref-6] Chen X, He W, Ye Z, Gai J, Lu W, Xing G (2024). Soybean seed pest damage detection method based on spatial frequency domain imaging combined with RL-SVM. Plant Methods.

[ref-7] Chiang HS, Talekar NS (1980). Identification of source of resistance to the bean fly and two other agromyzid flies in soybean and mungbean. Journal of Economic Entomology.

[ref-8] Chowdary KS, Harika R, Katlam BP (2024). Biological and morphometric characteristics of *Spodoptera litura* (*Fabricius*) on soybean under laboratory conditions. Uttar Pradesh Journal of Zoology.

[ref-9] Faiz MF, Hidayat P, Winasa IW, Guntoro D (2021). Effect of soybean leaf trichomes on the preference of various soybean pests on field. IOP Conference Series: Earth and Environmental Science.

[ref-10] Fattah A, Hannan MFI, Yasin M, Harnowo D, Nugraha Y, Wulanningtyas HS, Andriyani I (2024). The characteristics of several varieties and the effect of cropping management design on the level of pest damage and seed yield of soybeans in rainfed lowland rice fields. Frontiers in Sustainable Food Systems.

[ref-11] Gandham K, Gautam M, George J, Reddy GV, Kariyat R (2024). Muffled olfactory and sensory cues from the reproductive stage soybean selectively reduce oviposition of a major polyphagous herbivore, fall armyworm (*Spodoptera frugiperda*). Pest Management Science.

[ref-12] Gontijo PC, Abbade Neto DO, Oliveira RL, Michaud JP, Carvalho GA (2018). Non-target impacts of soybean insecticidal seed treatments on the life history and behavior of *Podisus nigrispinus*, a predator of fall armyworm. Chemosphere.

[ref-13] Hamawaki OT, de Sousa LB, Romanato FN, Nogueira APO, de Sousa CJr, Polizel AC (2012). Genetic parameters and variability in soybean genotypes. Comunicata Scientiae.

[ref-46] Hanafiah KA (2010). Rancangan percobaan.

[ref-14] Houngbo S, Zannou A, Aoudji AK, Sossou HC, Sinzogan AA, Sikirou R, Zossou E, Vodounon HS, Adomou AC, Ahanchede A (2020). Farmers’ knowledge and management practices of fall armyworm, *Spodoptera frugiperda* (*J.E. Smith*) in Benin, West. Agriculture.

[ref-15] Huang F (2020). Resistance of the fall armyworm, *Spodoptera frugiperda*, to transgenic *Bacillus thuringiensis* Cry1F corn in the Americas: lessons and implications for Bt corn IRM in China. Insect Science.

[ref-16] Huang L, Xue F, Wan J, Tang J, Liang Y, He H (2023). Effects of food plants on life-history traits of the newly invasive fall armyworm *Spodoptera frugiperda*. Journal of Applied Entomology.

[ref-17] Irshad SS, Panwar N, Bansal L, Thirumurugan S, Kumar S (2024). Plant resistance to insects in oilseed crops. Plant Resistance to Insects in Major Field Crops.

[ref-18] Jin T, Lin Y, Chi H, Xiang K, Ma G, Peng Z, Yi K (2020). Comparative performance of the fall armyworm (*Lepidoptera: Noctuidae*) reared on various cereal-based artificial diets. Journal of Economic Entomology.

[ref-19] Kanwal B, Tanwir S, Ahmad F, Ahmad JN (2024). Jasmonic acid and salicylic acid improved resistance against *Spodoptera frugiperda* infestation in maize by modulating growth and regulating redox homeostasis. Scientific Reports.

[ref-20] Kaur I, Watts S, Raya C, Raya J, Kariyat R (2022). Surface warfare: plant structural defenses challenge caterpillar feeding. Caterpillars in the Middle: Tritrophic Interactions in a Changing World.

[ref-21] Khan NA, Islam MS, Bhuiyan SH, Hasan KM, Hasan MK (2022). Evaluation of yield contributing characters and cluster analysis of soybean genotypes. Algerian Journal of Biosciences.

[ref-22] Lytra I, Evangelou V, Antonatos S, Georgopoulou I, Tselou E, Dimopoulou D, Arampatzis C, Milonas PG, Papachristos DP (2024). First data on the occurrence and population dynamics of the fall armyworm *Spodoptera frugiperda* (*Lepidoptera: Noctuidae*) in Greece. EPPO Bulletin.

[ref-23] Maurer TR, Tonello CZ, Machado BO, Rieder R, Suzana-Milan CS (2024). Controlling fall armyworm (*Spodoptera frugiperda*) in soybean: a comparative study. Caderno Pedagógico.

[ref-24] Nagoshi RN, Davis JA, Meagher RL, Musser FR, Head GP, Portillo HE, Terán H (2023). Investigating the migratory behavior of soybean looper, a major pest of soybean, through comparisons with the corn pest fall armyworm using mitochondrial haplotypes and a sex-linked marker. Genes.

[ref-25] Nandan R, Bandaru V, He J, Daughtry CS, Gowda PH, Suyker AE (2022). Evaluating optical remote sensing methods for estimating leaf area index for corn and soybean. Remote Sensing.

[ref-26] Patu BA, Sarjan M, Tarmizi T, Tantawizal T (2023). Agronomic characteristics of soybean production determination in two cultivation techniques attacked by *Etiella zinckenella* in dry land. AIP Conference Proceedings.

[ref-27] Prasad YG, Gayathri M, Sailaja V, Prabhakar M, Rao GR, Rekha G, Vennila S (2021). Evaluation of linear and nonlinear models for temperature-driven development of *Spodoptera litura* (*Fabricius*) on soybean crop. Journal of Agrometeorology.

[ref-28] Prasetyo WB, Hidayanto M (2016). Kajian ketahanan beberapa VUB kedelai terhadap serangan hama pemakan daun di Kabupaten Kutai Timur.

[ref-29] Santos-Alves D, Andrade-Carvalho G, Ferreira-Oliveira D, Duarte-Correa A (2018). Screening of Brazilian plant extracts as candidates for the control of *Spodoptera frugiperda* (Lepidoptera: Noctuidae). Revista Colombiana de Entomología.

[ref-30] Santos-Amaya OF, Tavares CS, Rodrigues JV, Oliveira EE, Guedes RN, Pereira EJ (2022). Strong fitness costs of fall armyworm resistance to dual-gene Bt maize are magnified on less-suitable host-crop cultivars. Agronomy.

[ref-31] Saritha R, Visalakshi M (2024). Legume-based profitable intercropping system for management of fall armyworm in maize. Legume Research.

[ref-32] Sarjan M, Fathiyani BD, Yassi A (2021). Attack intensity of sucking pods insect pest (*Riptortus linearis*) in different ages of soybean under drought stress conditions. IOP Conference Series: Earth and Environmental Science.

[ref-33] Sasane AR, Bhalkare SK, Rathod PK, Undirwade DB (2018). Biophysical basis of resistance in soybean genotypes against defoliators. Journal of Entomology and Zoology Studies.

[ref-34] Shafi I, Kariyat R (2025). Transgenerational imprints of sequential herbivory on soybean physiology and fitness traits. Plant-Environment Interactions.

[ref-35] Singh RK, Sreenivasulu N, Prasad M (2022). Potential of underutilized crops to introduce the nutritional diversity and achieve zero hunger. Functional & Integrative Genomics.

[ref-36] Sotelo-Cardona P, Chuang W, Lin M, Chiang M, Ramasamy S (2021). Oviposition preference not necessarily predicts offspring performance in the fall armyworm, *Spodoptera frugiperda* (*Lepidoptera: Noctuidae*) on vegetable crops. Scientific Reports.

[ref-37] Souza BH, Costa EN, Ribeiro ZA, Perlatti B, Cruz ME, Forim MR, Júnior AL, Stout MJ (2021). Soybean leaf age and plant stage influence expression of resistance to velvetbean caterpillar and fall armyworm. Chemoecology.

[ref-47] Syahrawati MY, Busniah M (2009). Serangga hama dan predator pada pertanaman kacang panjang (vigna sinensis (l.) savi ex has) fase generatif di kota padang. J. Pertanian.

[ref-38] Tao W, Zhang X, Zhang Y, Deng X, Zhang H, Zhang Z, Li Q, Jiang C (2024). Effects of the host plants of the maize-based intercropping systems on the growth, development and preference of fall armyworm, *Spodoptera frugiperda* (*Lepidoptera: Noctuidae*). Insects.

[ref-39] Thomas G, Tabanca N (2025). Harnessing chemical ecology for improved pest management-advances and future opportunities. Pest Management Science.

[ref-40] Thrash BC, Adamczyk J, Lorenz GM, Scott A, Armstrong JS, Pfannenstiel RS, Taillon N (2013). Laboratory evaluations of lepidopteran-active soybean seed treatments on survivorship of fall armyworm (*Lepidoptera: Noctuidae*) larvae. Florida Entomologist.

[ref-41] Udayakumar A, Shivalingaswamy TM, Bakthavatsalam N (2020). Legume-based intercropping for the management of fall armyworm, *Spodoptera frugiperda* L. in maize. Journal of Plant Diseases and Protection.

[ref-42] Wang Z, Zhu H, Jin D, Hou J, Liu X (2025). Effects of three different host plants on two sex life table parameters of the fall armyworm *Spodoptera frugiperda*. Scientific Reports.

[ref-43] Yadav M, Singh A (2024). Reprogramming of *Glycine max* (soybean) proteome in response to *Spodoptera litura* (common cutworm)-infestation. Journal of Plant Growth Regulation.

[ref-44] Yifei Z, Yang D, Guijun W, Bin L, Guangnan X, Fajun C (2018). Effects of elevated CO2 on plant chemistry, growth, yield of resistant soybean, and feeding of a target lepidoptera pest, *Spodoptera litura* (Lepidoptera: Noctuidae). Environmental Entomology.

[ref-45] Zhang YF, Wan GJ, Liu B, Zhang XG, Xing GN, Chen FJ (2018). Elevated CO2 and temperature alter development and food utilization of *Spodoptera litura* fed on resistant soybean. Journal of Applied Entomology.

